# A Bayesian Outlier Criterion to Detect SNPs under Selection in Large Data Sets

**DOI:** 10.1371/journal.pone.0011913

**Published:** 2010-08-02

**Authors:** Mathieu Gautier, Toby Dylan Hocking, Jean-Louis Foulley

**Affiliations:** 1 INRA, UMR1313 GABI, Jouy-en-Josas, France; 2 INRA, UMR1031 CBGP, Montferrier-sur-Lez, France; University of Manchester, United Kingdom

## Abstract

**Background:**

The recent advent of high-throughput SNP genotyping technologies has opened new avenues of research for population genetics. In particular, a growing interest in the identification of footprints of selection, based on genome scans for adaptive differentiation, has emerged.

**Methodology/Principal Findings:**

The purpose of this study is to develop an efficient model-based approach to perform Bayesian exploratory analyses for adaptive differentiation in very large SNP data sets. The basic idea is to start with a very simple model for neutral loci that is easy to implement under a Bayesian framework and to identify selected loci as outliers via Posterior Predictive P-values (PPP-values). Applications of this strategy are considered using two different statistical models. The first one was initially interpreted in the context of populations evolving respectively under pure genetic drift from a common ancestral population while the second one relies on populations under migration-drift equilibrium. Robustness and power of the two resulting Bayesian model-based approaches to detect SNP under selection are further evaluated through extensive simulations. An application to a cattle data set is also provided.

**Conclusions/Significance:**

The procedure described turns out to be much faster than former Bayesian approaches and also reasonably efficient especially to detect loci under positive selection.

## Introduction

The recent advent of high-throughput Single Nucleotide Polymorphism (SNP) genotyping technologies has opened new avenues of research for population genetics. In particular, a growing interest in the identification of footprints of selection, based on genome scans for adaptive differentiation, has emerged. Indeed, such approaches early proposed in the population genetics literature [1]–[5], look particularly relevant when studying populations belonging to a same species but adapted to different environmental conditions. However, their application to whole genome scan data mainly relied on the analysis of simple descriptive summary statistics, generally related to standard estimators of marker-specific *F_ST_*, used to investigate the variability of allele frequencies at different loci across and within populations. Markers affected by selection are then expected to display an unexpectedly high or low value relative to the null distribution of *F_ST_* for markers not under selection. This null distribution typically depends on the (usually unknown) demographic history of the populations surveyed and two main types of strategies have been reported to estimate it, using either *i)* data simulated under demographic models [6] which are generally simple and restrictive or *ii)* directly from the observed data under the assumption that most of the analyzed markers are neutral [7]–[9]. This latter empirical approach has become very popular because it is easy and fast to implement. However, its robustness and its power are difficult, if not impossible, to evaluate.

Alternatively, using simple demographic models, likelihood-based approaches allowing a full use of the information contained in the data sets have also been developed to distinguish, among the evolutionary forces shaping differences in allele frequency, those pertaining to population-specific factors (*e.g.* migration or drift) from those due to locus-specific factors (such as selection). Hence, relying on an infinite Wright island model with drift and migration at equilibrium, Beaumont and Balding [10] proposed a Bayesian modeling of allele frequencies involving both a “locus” and a “population” effects on genetic differentiation. Using this model, the efficiency to detect non-neutral loci has recently been further investigated through model choice strategies via Reversible Jump Markov Chain Monte Carlo (RJ-MCMC) algorithms [11], or by introducing locus-specific selection variables [12], [13]. Although the application of the latter approach to a large data set comprising 36,320 SNPs genotyped on 9 West African cattle populations illustrated its feasibility [12], the estimation of the posterior distributions for the parameters of interest remains computationally intensive due to model complexity and to no parallelizable Markov Chain Monte Carlo (MCMC) algorithms. Similarly and more recently, Guo *et al.* [14] investigated Bayesian hierarchical models to estimate locus-specific effects on *F_ST_*, statistical outliers being detected based on the Kullback-Leibler divergence measure between the posterior distributions of locus-specific effects and the common *F_ST_*.

The purpose of this study is to develop an efficient likelihood-based approach to perform Bayesian exploratory analyses for adaptive differentiation for very large SNP data sets. The basic idea is to start from a very simple model for neutral loci that will be easy to implement under a Bayesian framework and to identify selected loci as outliers via posterior predictive P-values (PPP-values) [15], [16]. We investigate two different statistical models: *i)* a model interpreted in the context of populations evolving under pure genetic drift from a common ancestral population [17] and *ii)* a model interpreted in the context of populations under migration-drift equilibrium [10], [18]. Robustness and power of both resulting classifiers are further evaluated and compared to a previous well described classifier [10]–[13] through extensive simulations and an application to the cattle data set mentioned previously.

## Methods

### The models

Let *x_ij_* be the observed reference allele (defined arbitrarily for instance by randomly choosing it as the ancestral or derived allele) count in population *j* (1≤*j*≤*J*) at the (bi-allelic) SNP *i* (1≤*i*≤*I*). The conditional distribution of *x_ij_* given the true allele frequency *α_ij_* is assumed to be binomial with parameters *n_ij_* (twice the number of genotyped individuals in population *j* at locus *i*) and *α_ij_*:
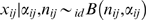
(1)Note that 1) implicitly assumes that populations are in Hardy-Weinberg Equilibrium (HWE) or equivalently their respective inbreeding coefficients (*F_IS_*) are null. Non null *F_IS_* could be taken into account in the model by considering instead that the three possible genotypes are drawn from a multinomial distribution with parameters corresponding to the number of individuals genotyped and genotype probabilities [19]. Nevertheless, for co-dominant markers such as SNPs and given the usual range of *F_IS_* values, the binomial distribution is fairly reasonable [12], [19].

In the first model considered (denoted model 1 hereafter) and according to Nicholson *et al.* [17], the second step assumes that the *α_ij_* are sampled from a truncated Gaussian distribution on the (0,1) segment
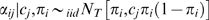
(2)plus additional probability masses at 0 and 1.

This distribution was proposed by Nicholson *et al.* [17] in the context of a pure-drift demographic model. In (2), the parameter *π_i_* stands for the allele frequency in the population ancestral to the *J* surveyed populations (assuming a star shaped phylogeny) and *c_j_* is a measure of differentiation of population 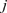. The probability masses at 0 and 1 aim at taking into account possible allele fixation due to genetic drift within a population. In Nicholson *et al.*'s model, these masses are conveniently defined as the lower (below 0) and upper (larger than 1) tail areas under the untruncated form of the Gaussian in (2) i.e.
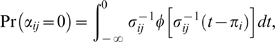
(3)

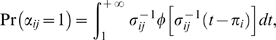
(4)where 
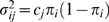
 and 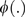 is the density of the 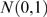 standard Gaussian.

The last level of the hierarchy corresponds to the distributions of *π_i_* and 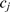. These two hyper-parameters are classically assumed to be sampled from *Beta* distributions:
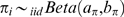
(5)

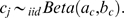
(6)In practice, the model was found to be robust to the parameter values of these *Beta* distributions [17]. We thus chose *a_π_ = b_π_* = 0.7 and *a_c_ = b_c_* = 1, leading to uniform prior distributions on 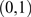 for *c_j_*.

Note that the introduction of the truncation (equations 2, 3 and 4) leads to some difficulty in the implementation of a MCMC algorithm in particular when defining the proposal distribution for the *α_ij_*. As suggested by G. Nicholson (personal communication) model 1 was considered as equivalent to the following one in which the first two levels are modified as
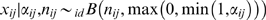
(7)

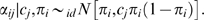
(8)In fact, the hierarchical model in (7) and (8) can be implemented equivalently by using a proxy variable 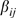 distributed as a regular Gaussian distribution 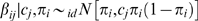 with the relationship 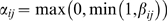.

The second model considered (model 2 hereafter) is similar to model 1 except that it assumes the *α_ij_* are sampled from a *Beta* distribution:
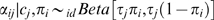
(9)where 
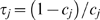
.

Note that model 2 does not consider the possibility of allele fixation since the Beta probability density function is either null or not defined in 0 and 1. Hence, alleles fixed in some (or all) populations are interpreted as being the result of a binomial sampling with a probability parameter close (but not equal) to 0 or 1. Demographic interpretation of this distribution on allele frequencies relies on an infinite Wright island model involving drift and migration at its equilibrium state [10], [18]. Under both model 1 (if we neglect truncature) and model 2, *c_j_* represents a scale parameter of the allele frequency variance and might thus be interpreted as a population specific *F_ST_* [17], [18].

For each model, we implemented a Metropolis-Hastings within Gibbs sampler to estimate the posterior distributions of the parameters of interest (Text S1). Program executables are available upon request from the first author. To check each program, we initially analyzed data sets simulated under the corresponding inference model (Figure S1). In addition, several data sets (including a real one) were also analyzed using a mirror version of the algorithms programmed in BUGS code and implemented in the OpenBUGS software [20]. For each model, results obtained with the two implementations were found in almost perfect agreement (data not shown).

### Decision criteria

Under the assumption of exchangeability among SNPs (*i.e.* neutrality), *c_j_* parameters are expected to be the same over SNPs within each population. Therefore, non neutral loci might be viewed as outliers with respect to the null model. One simple way to identify such loci thus consists of evaluating a local assessment of the null model (either model 1 or model 2) at each locus using Posterior Predictive Check tools. This can be easily accomplished by computing PPP-values which are the Bayesian counterparts of the frequentist P-values [15].

The PPP value for SNP *i* over the *J* populations is defined as
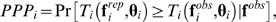
(10)where 
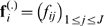
 with 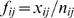 is the (reference) allele frequency of SNP *i* in population *j* and 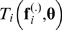 is a discrepancy criterion applied to replicated (*rep*) and observed (*obs*) data respectively given the values of model parameters 
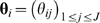
 with 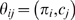. Notice that the probability that 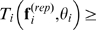 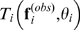 takes into account both variability in the replicates and uncertainty in the unknown parameters by integrating out with respect to these two sources of the variation via the distributions of 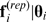 and of 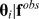 given all data 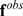 observed.

A first issue is to choose the discrepancy criterion which, contrarily to usual statistics, depends generally both on data and parameters. Here, we relied on a Chi-square type criterion ([21], formula 6.4, page 175)
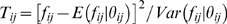
(11)with 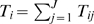.

It can be shown (Text S2) that for both models: 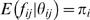 and 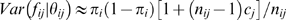. The null hypothesis being primarily based on the exchangeability assumptions between loci made at the second level of the hierarchy (formulae 2 and 9), we used here the moments of the marginal distribution of the 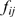
's as our measure of discrepancy between the data and the model. Letting the indicator variable 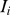 be equal to 1 if 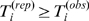 and 0 otherwise, then the corresponding 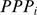 is simply the posterior expectations of 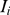 and can be easily computed from the Gibbs sampling outputs. The replicated data 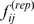 are generated at each iteration from the predictive distribution of 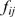 given each current values of 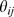.

Extreme probabilities at a given locus will indicate that the data at this locus are inconsistent with the model. Actually, small values correspond to positive selection and large values to balancing selection.

### Analyses under the Beaumont and Balding model

Under this model, referred hereafter as model 3, allele count data are modeled according to the reparameterized extension, recently proposed by Riebler *et al.* [13], of the Bayesian hierarchical model developed by Beaumont and Balding [10]. Model 3 is actually identical in its first levels to model 2 described above. Nevertheless, the differentiation parameter (*c_ij_*) is considered as both locus and population specific. In that respect, model 2 might be viewed as model 3 under the null hypothesis of neutrality (locus exchangeability). Hence, model 3 assumes the *α_ij_* are sampled from a *Beta* distribution:
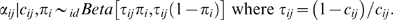
The *τ_ij_*'s are subsequently modeled via a linear model on the logistic transformation of the *c_ij_*. Since *c_ij_*/(1−*c_ij_*) = 1/*τ_ij_*, we can write this model in terms of:
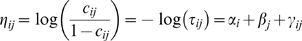
where *α_i_* is a locus effect, *β_j_* is a population effect and *γ_ij_* an error term corresponding to a departure of the logit of *c_ij_* from the additive decomposition. Following the Bayesian hierarchical structure of this model, additional levels of the model 3 are implemented as follows [10], [12], [13]: 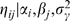
∼*_iid_* 
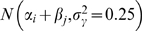
 with 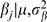
∼*_iid_* 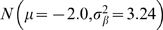 and 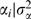
∼*_iid_* 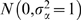. Recently, Riebler *et al.* [13] introduced in the above logistic model an auxiliary indicator variable *δ_i_* attached to each locus specifying whether it can be regarded as selected (*δ_i_* = 1) or neutral (*δ_i_* = 0). Under this reparameterized model, the previous parameters *α_i_* are written as: *α_i_* = δ_i_α_i_^*^ where 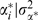
∼*_iid_* 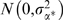. The model further assumes a Bernoulli distribution for the indicator *δ_i_* variable with parameter *P*: *δ_i_*|*P*∼Bin(1,*P*). *P* is itself assumed to be Beta distributed: *P∼Beta(0.2,1.8)* [12], [13]. Hence, by construction 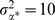.

The posterior distributions of the different parameters of interest were estimated via MCMC procedures as previously described [13] from 2,000 post burn-in samples (with a burn-in period of 2,500 iterations) and a thinning interval of k = 25. The decision rule to identify loci subjected to selection was based on a Bayes Factor (*BF*) derived at each locus from the posterior distribution of the *δ_i_* [12]. To make interpretation of the *BF* easier, we expressed it in deciban units (dB) *ie dB_i_ = 10log_10_(BF_i_)* [12].

### Simulations under neutrality

Four different demographic scenarios were investigated to evaluate the distribution of the PPP-values for neutrally evolving SNPs. In the first and second scenarios, allele count data were simulated for *L* = 1,000 independent bi-allelic neutral SNPs in *P* random mating populations evolving under a pure-drift Wright-Fisher demographic model over *T* non overlapping discrete generations from a common ancestral population. Under this model we thus expect the population specific *F_ST_* for a population with a constant (diploid) size *N* since divergence to be equal to 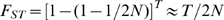. The same forward simulator as the one described below (Text S3) was used to generate data. In the first scenario (PDN1), P = 10 populations each of a constant (diploid) size *N = 500* were simulated from a common ancestral population with a star shaped phylogeny. To evaluate the effect of population hierarchical structure, in the second scenario (PDN2), *P* = 4 populations (*N = 500*) were initially (*T = 0*) simulated and two of these split in two populations (of size *N* = 250) at T = 30 and T = 50 generations respectively resulting in P = 5 populations for 30<T<50 and *P* = 6 populations for T>50. In the third scenario (MDN), allele count data were also simulated for *L* = 1,000 independent bi-allelic neutral SNPs in *P* random mating populations evolving under a Wright Fisher model with migration. Because in this latter case (and for neutral loci), the expected distribution of allele frequency at equilibrium corresponds to the one described above for model 2, data were simply simulated under the inference model 2.

Finally, to investigate a more complex (and realistic) demographic scenario, we simulated a data set under the calibrated model featuring the best-fitting conditions for four human populations (Europeans, Africans, Asians and African Americans) using the cosi package [22]. The demographic history of the populations corresponded to an Out-of-Africa model of an ancestral population that splits into Africans and non-Africans, and then into Europeans and Asians. African Americans are modeled as a recent admixture of the African and European populations. Fifty 250 kb long (autosomal) segments were simulated using heterogeneous recombination rates (picked from the empirical distribution of the deCODE genetic map [23]) leading to a total of 107,158 SNPs.

### Simulations under pure-drift and migration-drift demographic models with selection

Allele count data were simulated for *L* independent bi-allelic SNPs in *P* random mating populations evolving over T discrete non overlapping discrete generations from a common ancestral population (star shaped phylogeny). We first considered a simple pure-drift Wright-Fisher model (Text S3) in which the current populations are derived from an ancestral one in complete isolation (*i.e.* without migration between populations). We also simulated data under a simple Wright Fisher model with migration (Appendix III2) using a simplified version of previously described algorithms [10], [13]. In both models, selection was further introduced in the model by attaching a selective coefficient 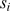 (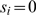 for a neutral locus) and a selection type (either positive or balancing) to each SNP *i*.

In all the simulations we did not consider mutation. In addition, SNP fixed for the same allele in all populations were discarded from further analysis. This might somewhat mimic part of the ascertainment bias expected in real data sets, since monomorphic SNPs are not expected to be genotyped or analyzed [12].

## Results

### PPP-value distribution under the null hypothesis

Under the hypothesis of exchangeability of the SNP (*i.e.* SNP neutrality), PPP-values for neutral loci are expected to be close to 0.5. However statistical noise introduces some dispersion around this value and thus some thresholds are necessary to define outliers. In addition, both models of differentiation could only be related to very simple demographic models and thus departure from the true demographic history might also affect the PPP-value distribution. Thus, in order to evaluate the robustness of both PPP-value classifiers based on the two alternative Bayesian hierarchical models 1 and 2, we first investigated the PPP-values distribution for data sets simulated under various neutral demographic scenarios (see Materials and Methods).

The first scenario (PDN1) is a simple Wright-Fisher pure drift model with 1,000 (neutral) SNPs segregating in 10 different populations of constant size originating from a common ancestral one *T* generations ago. As mentioned above, the statistical model 1 was proposed to deal with this latter kind of non-equilibrium demographic scenarios [17], [18]. Several values of *T* were considered to evaluate the effect of the level of differentiation: from *T = 10* (*F_ST_* = 0.01) to *T = 300* (*F_ST_* = 0.3). As detailed in Table 1, both statistical models resulted in these cases in average PPP-value close to 0.5 although model 1 tended to give average values lower than 0.5 as the differentiation increased. Interestingly, the dispersion (as measured by the standard deviation) decreases with differentiation. Hence, under this demographic model, the probability of detecting false positives SNPs (*i.e.* truly neutral SNPs with extreme PPP-values) will decrease with the level of differentiation, model 1 being less robust than model 2. For instance no SNP displayed a PPP-value below 0.1 when *T*>80 (*F_ST_*>0.08) for model 1 and when *T*>40 with model 2 (*F_ST_*>0.04). Similarly and for both models, no SNPs displayed a PPP-value above 0.9 when T>40 (*F_ST_*>0.04). The lack of reliability of both models at low level of divergence might be partly explained by a clear underestimation of population specific *F_ST_* at low level of divergence (*F_ST_<0.05*) (Figure S2). This tendency towards underestimation was not observed when simulating data under either inference model 1 or 2 (Figure S1) indicating an imperfect fit of low level of pure drift divergence. In addition, we also noticed that for very high level of divergence (*F_ST_>0.4*), estimates were strongly upwardly biased, especially in the case of model 1. Thus these two models didn't appear relevant for such high level of pure drift divergence as expected by the marked difference (even for SNPs with ancestral reference allele frequency close to 0.5) between the expected distribution of within population allele frequency [24] and the distributions assumed in the models (Figure S3).

**Table 1:** Parameters of the distribution of the PPP-values obtained with model 1 and model 2 under the null hypothesis.

	model 1				model 2			
MODEL (#pop. - #SNPs)	Mean (sd)	Min-Max	%<0.05 (0.1–0.2)	%>0.95 (0.9–0.8)	Mean (sd)	Min-Max	%<0.05 (0.1–0.2)	%>0.95 (0.9–0.8)
PDN1 (10–1000) : T = 10 ; *F_ST_* = 0.01	0.516 (0.27)	0.002–1.000	2.90 (6.70–15.4)	2.20 (8.10–18.4)	0.520 (0.27)	0.002–0.981	2.40 (6.40–14.8)	1.90 (7.30–19.0)
PDN1 (10–1000) : T = 20 ; *F_ST_* = 0.02	0.490 (0.24)	0.011–0.970	1.00 (3.40–13.6)	0.70 (3.80–11.7)	0.497 (0.24)	0.028–0.979	0.60 (2.30–11.7)	1.00 (4.00–12.2)
PDN1 (10–1000) : T = 30 ; *F_ST_* = 0.03	0.478 (0.21)	0.032–0.968	0.30 (1.60–9.20)	0.10 (1.10–7.40)	0.498 (0.20)	0.073–0.962	0.00 (0.50–6.20)	0.20 (1.30–8.30)
PDN1 (10–1000) : T = 40 ; *F_ST_* = 0.04	0.475 (0.19)	0.045–0.921	0.10 (1.40–7.40)	0.00 (0.70–4.70)	0.502 (0.18)	0.052–0.934	0.00 (0.30–3.40)	0.00 (0.50–5.40)
PDN1 (10–1000) : T = 50 ; *F_ST_* = 0.05	0.470 (0.17)	0.054–0.925	0.00 (0.20–4.00)	0.00 (0.10–2.50)	0.502 (0.16)	0.130–0.923	0.00 (0.00–1.50)	0.00 (0.30–3.90)
PDN1 (10–1000) : T = 80 ; *F_ST_* = 0.08	0.468 (0.14)	0.030–0.884	0.10 (0.20–2.70)	0.00 (0.00–0.90)	0.509 (0.13)	0.150–0.899	0.00 (0.00–0.30)	0.00 (0.00–1.10)
PDN1 (10–1000) : T = 100 ; *F_ST_* = 0.1	0.461 (0.13)	0.109–0.885	0.00 (0.00–1.80)	0.00 (0.00–0.20)	0.506 (0.11)	0.171–0.841	0.00 (0.00–0.10)	0.00 (0.00–0.50)
PDN1 (10–1000) : T = 150 ; *F_ST_* = 0.15	0.460 (0.11)	0.175–0.806	0.00 (0.00–0.30)	0.00 (0.00–0.10)	0.510 (0.09)	0.262–0.821	0.00 (0.00–0.00)	0.00 (0.00–0.20)
PDN1 (10–1000) : T = 200 ; *F_ST_* = 0.2	0.459 (0.09)	0.226–0.848	0.00 (0.00–0.00)	0.00 (0.00–0.10)	0.513 (0.08)	0.290–0.802	0.00 (0.00–0.00)	0.00 (0.00–0.10)
PDN1 (10–1000) : T = 300 ; *F_ST_* = 0.3	0.465 (0.07)	0.284–0.734	0.00 (0.00–0.00)	0.00 (0.00–0.00)	0.516 (0.07)	0.317–0.783	0.00 (0.00–0.00)	0.00 (0.00–0.00)
PDN2 (4–1000) : T = 30	0.490 (0.19)	0.062–0.864	0.00 (0.60–6.40)	0.00 (0.00–3.80)	0.498 (0.18)	0.079–0.881	0.00 (0.30–4.80)	0.00 (0.00–3.70)
PDN2 (5–1000) : T = 35	0.501 (0.20)	0.039–0.867	0.30 (1.50–7.70)	0.00 (0.00–4.10)	0.509 (0.19)	0.045–0.881	0.10 (0.40–5.00)	0.00 (0.00–4.30)
PDN2 (5–1000) : T = 50	0.483 (0.16)	0.137–0.867	0.00 (0.00–1.90)	0.00 (0.00–2.20)	0.502 (0.15)	0.159–0.879	0.00 (0.00–0.30)	0.00 (0.00–2.30)
PDN2 (6–1000) : T = 55	0.496 (0.18)	0.084–0.882	0.00 (0.10–5.10)	0.00 (0.00–3.10)	0.516 (0.17)	0.107–0.874	0.00 (0.00–1.40)	0.00 (0.00–3.50)
PDN2 (6–1000) : T = 60	0.485 (0.15)	0.147–0.853	0.00 (0.00–2.40)	0.00 (0.00–1.40)	0.509 (0.14)	0.126–0.869	0.00 (0.00–0.70)	0.00 (0.00–1.60)
PDN2 (6–1000) : T = 70	0.478 (0.14)	0.163–0.858	0.00 (0.00–1.10)	0.00 (0.00–0.90)	0.505 (0.13)	0.173–0.855	0.00 (0.00–0.10)	0.00 (0.00–0.90)
PDN2 (6–1000) : T = 100	0.474 (0.11)	0.226–0.802	0.00 (0.00–0.00)	0.00 (0.00–0.10)	0.505 (0.10)	0.227–0.817	0.00 (0.00–0.00)	0.00 (0.00–0.10)
PDN2 (6–1000) : T = 150	0.473 (0.08)	0.280–0.765	0.00 (0.00–0.00)	0.00 (0.00–0.00)	0.504 (0.08)	0.265–0.789	0.00 (0.00–0.00)	0.00 (0.00–0.00)
MDN (8–1000) : *F_ST_* = 0.01	0.483 (0.22)	0.030–0.957	0.60 (3.10–11.2)	0.40 (2.60–9.90)	0.494 (0.22)	0.034–0.962	0.10 (1.70–9.30)	0.40 (2.50–10.1)
MDN (8–1000) : *F_ST_* = 0.025	0.473 (0.20)	0.027–0.930	0.20 (2.10–8.30)	0.00 (0.40–5.20)	0.498 (0.19)	0.065–0.930	0.00 (0.20–4.10)	0.00 (0.50–7.10)
MDN (8–1000) : *F_ST_* = 0.05	0.461 (0.17)	0.074–0.915	0.00 (0.20–4.50)	0.00 (0.10–2.80)	0.500 (0.15)	0.120–0.931	0.00 (0.00–0.70)	0.00 (0.10–3.00)
MDN (8–1000) : *F_ST_* = 0.075	0.451 (0.14)	0.091–0.899	0.00 (0.40–3.40)	0.00 (0.00–0.90)	0.501 (0.12)	0.160–0.920	0.00 (0.00–0.20)	0.00 (0.10–0.80)
MDN (8–1000) : *F_ST_* = 0.1	0.448 (0.13)	0.119–0.875	0.00 (0.00–2.20)	0.00 (0.00–0.40)	0.499 (0.11)	0.187–0.875	0.00 (0.00–0.10)	0.00 (0.00–0.50)
MDN (8–1000) : *F_ST_* = 0.15	0.445 (0.11)	0.184–0.817	0.00 (0.00–0.10)	0.00 (0.00–0.10)	0.502 (0.09)	0.237–0.780	0.00 (0.00–0.00)	0.00 (0.00–0.00)
MDN (8–1000) : *F_ST_* = 0.2	0.438 (0.09)	0.245–0.848	0.00 (0.00–0.00)	0.00 (0.00–0.10)	0.498 (0.08)	0.261–0.741	0.00 (0.00–0.00)	0.00 (0.00–0.00)
COA (4–107158): all	0.480 (0.14)	0.011–0.843	0.10 (0.30–2.90)	0.00 (0.00–0.10)	0.496 (0.13)	0.074–0.819	0.00 (0.00–0.50)	0.00 (0.00–0.00)
COA (4–32559): all*	0.515 (0.16)	0.016–0.862	0.10 (0.60–3.40)	0.00 (0.00–0.50)	0.521 (0.14)	0.055–0.885	0.00 (0.10–1.30)	0.00 (0.00–0.30)
COA (3–94321): EuroAfamAfri	0.479 (0.15)	0.015–0.856	0.10 (0.40–3.60)	0.00 (0.00–0.10)	0.480 (0.14)	0.020–0.851	0.00 (0.30–3.00)	0.00 (0.00–0.00)
COA (3–58026): EuroAfamAfri*	0.517 (0.17)	0.007–0.884	0.30 (1.30–6.20)	0.00 (0.00–0.60)	0.527 (0.16)	0.006–0.879	0.10 (0.80–4.70)	0.00 (0.00–0.30)
COA (3–96797): EuroAfamAsia	0.455 (0.07)	0.189–0.762	0.00 (0.00–0.00)	0.00 (0.00–0.00)	0.478 (0.07)	0.172–0.752	0.00 (0.00–0.00)	0.00 (0.00–0.00)
COA (3–34318): EuroAfriAsia*	0.478 (0.11)	0.191–0.760	0.00 (0.00–0.00)	0.00 (0.00–0.00)	0.500 (0.11)	0.116–0.778	0.00 (0.00–0.40)	0.00 (0.00–0.00)

Several data sets consisting of neutral SNPs evolving under four different demographic scenarios were simulated. PDN1 correspond to a pure-drift demographic model with a star shaped phylogeny (10 populations originating from a common ancestral one, T generations ago). PDN2 corresponds to a pure-drift demographic model with a more complex history starting with 4 populations at *T* = 0, two of which giving rise to 2 populations at T = 30 and T = 50 generations respectively. MDN corresponds to a migration-drift model with 10 populations (inference model 2). Finally, COA corresponds to the calibrated Out-of-Africa model (an ancestral population splits into Afri = Africans and non-Africans, and then into Euro = Europeans and Asia = Asians) featuring the best-fitting conditions for four human populations (Euro, Afri, Asia, and AfAm = African Americans that are modeled as a recent admixture of Afri and Euro) [22]. Based on the complete data sets, three different groups were considered (all = Euro, AfAm, Afri and Asia; EuroAfAmAfri = Euro, AfAm and Asia; EuroAfriAsia = Euro, Afri and Asia) and two analyses per group were carried out (either with all SNPs segregating in at least one populations or only SNPs with a MAF>0.01 in at least two populations (*)).

Under such pure-drift divergence models, a strong (and implicit) hypothesis which might often be violated concerns the star shaped phylogeny relating the different populations. As an attempt to evaluate consequences of departure from such simple phylogeny, we thus analyzed data sets simulated under a pure-drift demographic scenario with a more complex history (PDN2) starting with 4 populations at *T* = 0, two of which giving rise to 2 populations at *T* = 30 and *T* = 50 generations respectively. This resulted in an increase of PPP-values dispersion soon after the population split (*e.g. T* = 35 and *T* = 55 in Table 1). This could be directly related to previous observations since population splits lead to a clear underestimation of population-specific *F_ST_* for the newly arisen populations (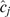 tending to 0 at early time after the split). Overall, although bias in the estimation of the 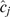 persisted, the dispersion decreased as the number of generations (since the split), thus rendering the PPP-value approach relatively robust.

In a third demographic scenario (MDN), we simulated 1,000 SNPs segregating in 10 populations under a migration-drift equilibrium which corresponds to the inference model 2 (see Methods). Although, no clear bias was observed in the estimation of the 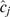 with both models (*e.g.* Figure S2), PPP-value dispersion increased as the level of differentiation decreased. Nevertheless and as expected, model 1 appeared less robust than model 2 at low level of differentiation (*F_ST_*<0.05). In addition, the departure of the average PPP-values toward values lower than (the expected) 0.5 appeared more pronounced as the differentiation increased.

We finally explored with coalescent simulations a more realistic scenario (COA) consisting in the calibrated Out-of-Africa model featuring the best-fitting conditions for four human populations (Europeans, Africans, Asians, and African Americans modelled as a recent admixture of Africans and Europeans) [22]. Note that because, 50 independent 250 kb segments were simulated, some SNPs were not independent. Based on the complete resulting data sets, three different population groups were analyzed (Table 1). Results were overall consistent with those reported above for more simple scenarios. Hence, for the EuroAfriAsia group, almost no SNP displayed PPP-values below 0.2 or above 0.8. As expected from previous observations on PDN2 simulated data sets, introducing the African American recently admixed populations lead to a higher dispersion of PPP-values (more pronounced for the analysis of the EuroAfAmAfri group) together with a low estimated 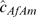 for this population (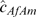
<0.015 when analyzing the four simulated populations and 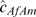
<0.001 when analyzing the EuroAfAmAfri group). Nevertheless, only a small proportion of the SNPs (<1%) displayed PPP-values lower than 0.1 or higher than 0.9.

On the basis of these different simulations, the distribution of PPP-values appears robust to various demographic scenarios provided that the global estimated population differentiation (average) is not too small (as a rule of thumb *F_ST_*>0.05). In addition and as previously mentioned [12], [17], the two models considered in this study remain almost insensitive to the prior distribution of the 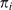 which might be (demographically) interpreted as the allele frequency in the ancestral population (under a pure-drift model) or in the gene pool (under a migration-drift model at equilibrium). As a result, the models are expected to be robust to the chosen SNP ascertainment scheme. Hence, for the first three scenarios investigated above, the distribution of PPP-values appeared almost unchanged when keeping all the SNPs in the analysis, even those fixed in all populations (data not shown). Similarly, results reported in Table 1 for a different SNP ascertainment scheme applied on the COA simulations which consisted in keeping only those SNPs segregating (MAF>0.01) in at least two populations suggested that the influence of ascertainment bias on the PPP-value distribution is small.

In order to evaluate to what extent selection causes an outlier PPP-value for the underlying SNPs, we analyzed several simulated data sets with some SNPs subjected both to balancing and positive selection (see Materials and Methods) and three different selection coefficients as representative of low (*s = 0.02*), moderate (*s = 0.05*) and high (*s = 0.10*) selection intensity.

### Analyzing data sets simulated under a pure drift demographic model with selection

We first considered a pure-drift demographic scenario similar to the PDN1 one described previously. We herein report results obtained with a data set consisting of eight populations with a constant haploid size of N = 500 deriving from a common ancestral one and genotyped for 10,000 SNPs among which 8,500 were neutral (*s_i_ = 0*), 750 were subjected to positive selection (250 with *s_i_ = 0.02*, 250 with *s_i_ = 0.05* and 250 with *s_i_ = 0.1*) and 750 were subjected to balancing selection (250 with *s_i_ = 0.02*, 250 with *s_i_ = 0.05* and 250 with *s_i_ = 0.1*). Five such data sets were generated with *T = 10*, *T = 25*, *T = 50*, *T = 75* and *T = 100* generations after divergence. We thus expected (assuming neutrality) for each population an *F_ST_* (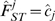) equal to 0.0198, 0.0488, 0.0953, 0.139 and 0.181 respectively (see Material and Methods). From moderate level of divergence (*T/N>0.1*), both models lead to a clear and increasing overestimation of *F_ST_*, the bias being more pronounced with model 1 than model 2 (Figure S4). Compared to data sets containing only neutral SNPs (see above and Figure S2), it appears that this overestimation was mostly related to the presence of SNPs subjected to (positive) selection. Nevertheless, for moderate level of divergence (roughly speaking when 0.05<*F_ST_*<0.2) estimation of *c_j_* appeared to be relatively robust to selection.

Interestingly, estimates of the ancestral reference allele frequency 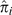 (mean of the posterior distribution of 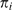) were remarkably consistent with their corresponding simulated values for neutral SNPs although precision decreased with increased level of divergence (Figure S5). Indeed, the correlation between simulated and estimated ancestral allele frequencies was always above 0.98 with both models, while the Root Relative Mean Square Error (RRMSE) ranged from 3.25% (*T = 10*) to 10.7% (*T = 100*) with model 1 and from 3.24% (*T = 10*) to 9.90% (*T = 100*) with model 2. However, these 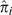 estimates were biased for SNPs under selection (Figure S5), the bias increasing with divergence and intensity of selection. More precisely, the RRMSE ranged from 4.04% (*T = 10* and *s = 0.02*) to 40.9% (*T = 100* and *s = 0.10*) with model 1 and from 4.04% to 39.5% with model 2 for SNPs under balancing selection. Similarly, for SNPs subjected to positive selection, the RRMSE ranged from 3.24% (*T = 10* and *s = 0.02*) to 41.4% (*T = 100* and *s = 0.10*) with model 1 and from 3.23% to 40.8% with model 2.

**Figure 1:**
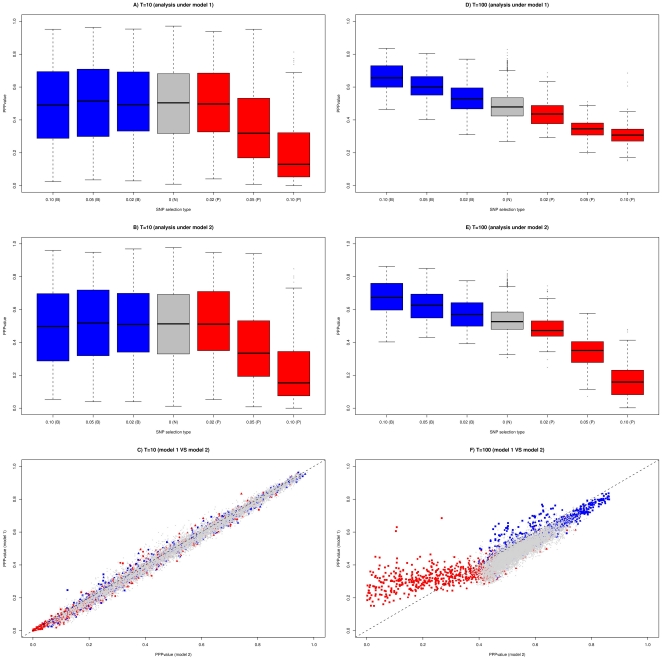
Distribution of the PPP-values estimates obtained with model 1 and model 2 for two data sets (*T = 10* and *T = 100*) simulated under a pure-drift demographic scenario. Each data set consists of genotyping data on 8 populations for 10,000 SNPs: 8,500 neutral SNPs (N), 3×250 SNPs subjected to positive selection (P) of varying intensity (*s = 0.02*, *s = 0.5* and *s = 0.10*) and 3×250 = 750 SNPs subjected to balancing selection (B) of varying intensity (*s = 0.02*, *s = 0.5* and *s = 0.10*). Boxplots of the PPP-values as a function of the type and intensity of selection are represented for data set simulated with *T = 10* and analyzed with model 1 (A) and model 2 (B) and for data set simulated with *T = 100* and analyzed with model 1 (D) and model 2 (E). C) and F) PPP-values estimated with model 1 are plotted against those estimated with model 2 for the data set simulated with *T = 10* and *T = 100* respectively. Neutral SNPs are plotted in grey while SNPs subjected to positive (respectively balancing) selection are plotted in red (respectively blue). In addition, the simulated coefficients of selection are represented by a triangle (*s = 0.02*), a circle (*s = 0.05*) or a square (*s = 0.10*).

For these five simulated data sets the mean of the different PPP-value distributions were always close to 0.5 (from 0.480 to 0.489 for model 1 results and from 0.501 to 0.528 for model 2 results) while the standard deviation decreased with level of divergence (from 0.235 when T = 10 to 0.106 when T = 100 for model 1 results and from 0.231 when T = 10 to 0.0958 when T = 100 for model 2 results). As expected and shown in Figure 1 for two of these simulated data sets (*T = 10* and *T = 100*), the PPP-value median (and mean) remained close to 0.5 for neutral SNPs while tending to 0 (respectively 1) for SNPs subjected to positive (respectively balancing) selection. Moreover, this trend was more pronounced as the selective coefficient and differentiation increased and for SNPs under positive selection. As a result, the tails were more enriched in SNPs under selection (Table S1), model 2 showing an increased power of discrimination compared to model 1 (at least for SNPs under positive selection). For instance, while 7.5% of the simulated SNPs were subjected to positive selection, this proportion ranged from 39.2% (*T = 10*) to 73.6% (when *T = 100*) for the 250 SNPs with the lowest PPP-values obtained with model 1 and from 40.4% (*T = 10*) to 100% (*T = 75* and *T = 100*) with model 2 (a vast majority of these SNPs being those with high value of *s*). Discrimination based on PPP-values appeared nevertheless far less efficient in identifying SNPs under balancing selection (Table S1). Hence, while 7.5% of the simulated SNPs were subjected to balancing selection, this proportion ranged from 15.6% (*T = 10*) to 64.0% (when *T = 75* and *T = 100*) for the 250 SNPs with the lowest PPP-values obtained with model 1 and from 9.20% (*T = 10*) to 56.0% (*T = 100*) with model 2.

**Figure 2:**
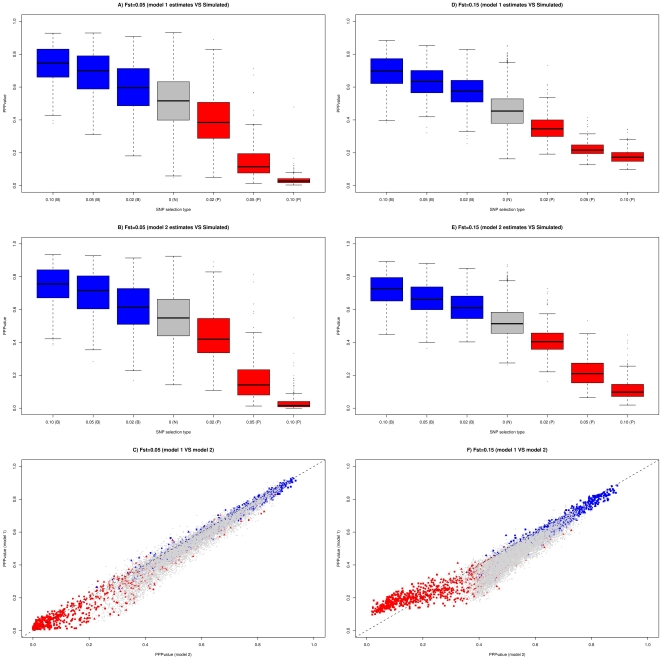
Distribution of the PPP-values estimates obtained with model 1 and model 2 for two data sets (*F_ST_ = 0.05* and *F_ST_ = 0.15*) simulated under a migration-drift demographic scenario. Each data set consists of genotyping data for 10,000 SNPs: 8,500 neutral SNPs (N), 3×250 SNPs subjected to positive selection (P) of varying intensity (*s = 0.02*, *s = 0.5* and *s = 0.10*) and 3×250 = 750 SNPs subjected to balancing selection (B) of varying intensity (*s = 0.02*, *s = 0.5* and *s = 0.10*). Boxplots of the PPP-values as a function of the type and intensity of selection are represented for data set simulated with *T = 10* and analyzed with model 1 (A) and model 2 (B) and for data set simulated with *T = 100* and analyzed with model 1 (D) and model 2 (E). C) and F) PPP-values estimated with model 1 are plotted against those estimated with model 2 for the data set simulated with *T = 10* and *T = 100* respectively. Neutral SNPs are plotted in grey while SNPs subjected to positive (respectively balancing) selection are plotted in red (respectively blue). In addition, the simulated coefficients of selection are represented by a triangle (*s = 0.02*), a circle (*s = 0.05*) or a square (*s = 0.10*).

Although we expected lower (resp. upper) tails to be enriched in SNPs under positive (resp. balancing) selection, identifying outliers on the observed PPP-value distribution makes it impossible, in practice, to control for False Discovery Rate (FDR) or False Negative Rate. We thus further investigated the power and robustness of each model, based on the simulated data sets, by computing FDR and recording the number of SNPs properly identified as subjected to selection for different PPP-value threshold (Table 2). For a given threshold the FDR but also the power decreased as the number of simulated generations increased, which was expected since this also resulted in sharpening the overall PPP-value distribution due in particular to an increase of the *c_j_* and thus the allele frequency variance. Similarly, the power was always higher when considering SNPs subjected to stronger selection (see above). Model 2 appeared far more efficient than model 1 mainly because PPP-value estimates were more extreme for SNPs under selection (*e.g.* Figures 2C and 2F). For instance, when *T = 75* a threshold of 0.2 to detect SNPs under positive selection resulted in a FDR equal to 0 while the power was equal to respectively 13.6% when using model 1 and 68.4% when using model 2. The associated FDR for such a threshold when T = 10 was close to 10% for both models (Table 1). Finally, performing similar analyses on simulated data sets with a lower number of populations (Tables 1 and 2) lead to only a slight decrease in overall power.

**Table 2:** Power and robustness of model 1 and model 2 to detect SNPs subjected to selection.

Simulation Model	Analysis	PPP<0.05	PPP<0.10	PPP<0.20	PPP<0.30	PPP>0.95	PPP>0.90	PPP>0.80	PPP>0.70
PDM (T = 10, J = 8)	Model 1	1/13/58 (0.8)	5/34/99 (3.2)	22/82/153 (11.4)	50/119/183 (22.8)	2/1/1 (0.3)	8/8/10 (2.7)	27/37/33 (11.5)	59/66/57 (22.6)
PDM (T = 10, J = 8)	Model 2	0/9/50 (0.2)	3/26/88 (1.9)	16/68/150 (9.4)	46/109/179 (21.1)	2/0/2 (0.2)	9/11/11 (3.2)	29/37/35 (12.3)	62/71/62 (23.9)
PDM (T = 25, J = 8)	Model 1	0/0/23 (0.01)	1/5/91 (0.3)	10/64/180 (3.1)	41/115/231 (13.2)	0/0/0 (0)	0/0/4 (0.1)	6/15/24 (3.0)	33/43/51 (11.3)
PDM (T = 25, J = 8)	Model 2	0/0/42 (0)	0/2/84 (0)	5/32/147 (0.3)	21/94/213 (4.9)	0/0/0 (0)	0/1/5 (0.1)	8/18/29 (4.1)	43/56/57 (15.2)
PDM (T = 50, J = 8)	Model 1	0/0/0 (0)	0/0/2 (0)	2/21/120 (0.5)	23/131/216 (5.8)	0/0/0 (0)	0/0/0 (0)	1/9/26 (0.3)	19/33/79 (4.2)
PDM (T = 50, J = 8)	Model 2	0/0/24 (0)	0/3/58 (0)	0/24/131 (0)	7/99/189 (0.3)	0/0/0 (0)	0/0/0 (0)	3/12/33 (0.6)	28/49/85 (7.7)
PDM (T = 75, J = 8)	Model 1	0/0/0 (0)	0/0/0 (0)	0/2/34 (0)	7/77/194 (2.5)	0/0/0 (0)	0/0/0 (0)	1/9/18 (0.1)	12/32/78 (1.5)
PDM (T = 75, J = 8)	Model 2	0/0/35 (0)	0/1/82 (0)	0/16/171 (0)	4/80/232 (0.01)	0/0/0 (0)	0/0/0 (0)	1/15/35 (0.2)	22/50/96 (4.0)
PDM (T = 100, J = 8)	Model 1	0/0/0 (0)	0/0/0 (0)	0/1/10 (0)	1/51/104 (0.5)	0/0/0 (0)	0/0/0 (0)	0/1/10 (0.02)	10/39/88 (0.7)
PDM (T = 100, J = 8)	Model 2	0/0/38 (0)	0/1/77 (0)	0/20/161 (0)	2/79/228 (0)	0/0/0 (0)	0/0/0 (0)	0/8/35 (0.1)	21/59/103 (2.4)
PDM (T = 10, J = 4)	Model 1	1/6/35 (0.5)	4/17/68 (2.3)	21/50/112 (9.6)	55/87/142 (20.3)	0/0/0 (0)	0/0/0 (0)	20/23/23 (8.0)	62/55/62 (21.7)
PDM (T = 10, J = 4)	Model 2	0/5/31 (0.3)	3/14/61 (1.7)	22/45/108 (8.9)	57/88/141 (19.7)	0/0/0 (0)	0/0/0 (0)	18/21/24 (8.5)	61/55/64 (22.7)
PDM (T = 25, J = 4)	Model 1	0/0/1 (0)	0/1/24 (0)	4/22/106 (1.3)	28/79/171 (9.1)	0/0/0 (0)	0/0/0 (0)	1/6/7 (1.2)	30/28/44 (9.9)
PDM (T = 25, J = 4)	Model 2	0/0/11 (0)	0/2/26 (0.01)	2/17/103 (0.3)	19/67/157 (5.3)	0/0/0 (0)	0/0/0 (0)	3/3/8 (1.2)	27/34/52 (11.5)
PDM (T = 50, J = 4)	Model 1	0/0/0 (0)	0/0/0 (0)	0/0/25 (0)	10/61/142 (2.5)	0/0/0 (0)	0/0/0 (0)	0/0/0 (0)	11/8/34 (3.3)
PDM (T = 50, J = 4)	Model 2	0/0/6 (0)	0/0/18 (0)	0/3/83 (0)	3/42/154 (0.4)	0/0/0 (0)	0/0/0 (0)	0/0/0 (0.01)	13/14/48 (3.7)
PDM (T = 75, J = 4)	Model 1	0/0/0 (0)	0/0/0 (0)	0/0/3 (0)	4/28/98 (0.4)	0/0/0 (0)	0/0/0 (0)	0/0/0 (0)	3/14/40 (1.3)
PDM (T = 75, J = 4)	Model 2	0/0/5 (0)	0/1/26 (0)	0/7/99 (0)	0/42/159 (0.1)	0/0/0 (0)	0/0/0 (0)	0/0/0 (0)	5/17/62 (1.5)
PDM (T = 100, J = 4)	Model 1	0/0/0 (0)	0/0/0 (0)	0/1/1 (0)	0/9/40 (0.02)	0/0/0 (0)	0/0/0 (0)	0/0/0 (0.012)	2/5/13 (0.5)
PDM (T = 100, J = 4)	Model 2	0/0/5 (0)	0/3/26 (0)	0/11/88 (0)	1/53/170 (0.1)	0/0/0 (0)	0/0/0 (0)	0/0/0 (0)	11/12/44 (0.612)
MDM (F_ST_ = 0.05, J = 8)	Model 1	0/14/201 (0.01)	3/103/241 (0.3)	22/192/249 (2.8)	71/222/249 (10.5)	0/0/0 (0)	1/7/6 (0.2)	20/56/89 (3.9)	70/123/158 (13.3)
MDM (F_ST_ = 0.05, J = 8)	Model 2	0/19/197 (0)	0/86/232 (0)	10/167/246 (0.2)	43/205/248 (3.4)	0/0/0 (0)	1/9/8 (0.2)	26/67/94 (5.4)	78/138/171 (18.1)
MDM (F_ST_ = 0.1, J = 8)	Model 1	0/0/0 (0)	0/3/46 (0)	7/158/241 (1.5)	72/237/250 (8.9)	0/0/0 (0)	0/0/3 (0)	9/17/55 (0.4)	43/78/140 (4.4)
MDM (F_ST_ = 0.1, J = 8)	Model 2	0/1/98 (0)	0/20/196 (0)	1/144/242 (0)	33/231/248 (0.4)	0/0/0 (0)	0/0/4 (0)	13/29/73 (0.9)	58/103/158 (8.5)
MDM (F_ST_ = 0.15, J = 8)	Model 1	0/0/0 (0)	0/0/1 (0)	2/78/182 (0.3)	63/237/247 (8.0)	0/0/0 (0)	0/0/0 (0)	2/15/39 (0.1)	29/64/122 (1.3)
MDM (F_ST_ = 0.15, J = 8)	Model 2	0/0/30 (0)	0/14/129 (0)	1/113/223 (0)	24/209/245 (0.1)	0/0/0 (0)	0/0/0 (0)	6/22/58 (0.1)	50/88/149 (3.9)

Results for thirteen different data sets are reported, ten of which were simulated under a pure drift demographic model (PDM) with a varying simulated number T of generations and J of populations; and three were simulated under a migration drift model (MDM) with a varying level of migration controlled by the simulated *F_ST_*. For each of the simulated data sets and analyses (either with model 1 or model 2), the numbers of SNPs subjected to selection, among the 250 simulated for each of the three classes of selection coefficient (*s = 0.02*/*s = 0.05*/*s = 0.10*), displaying a PPP-value below (for SNP subjective to positive selection) or above (for SNP subjective to balancing selection) the threshold considered are reported. In parenthesis is also given the estimated FDR in % (proportion of SNPs with a PPP-value below the threshold among the 8,500 neutral SNPs simulated).

### Analyzing data sets simulated under a migration drift demographic model with selection

We further evaluated both statistical models on data sets simulated under a migration drift demographic model (see Material and Methods). Note that assuming populations have reached (migration-drift) equilibrium, model 2 is consistent with such demographic scenarios (see Material and Methods). As in the previous section, we herein reported results obtained with data sets consisting of eight populations with a constant haploid size of *N = 500* which, according to a Wright island demographic model and during 250 generations, exchanged migrant alleles through a common gene pool (Text S3). For each data set, 10,000 SNPs were simulated among which 8,500 were neutral (*s_i_ = 0*), 750 were subjected to positive selection (250 with *s_i_ = 0.02*, 250 with *s_i_ = 0.05* and 250 with *s_i_ = 0.1*) and 750 were subjected to balancing selection (250 with *s_i_ = 0.02*, 250 with *s_i_ = 0.05* and 250 with *s_i_ = 0.1*). The proportion of migrant alleles arriving and leaving each population was further controlled by simulated population specific *F_ST_* values which, for simplicity, were set equal in each population. Three data sets were generated with *F_ST_ = 0.05*, *F_ST_ = 0.10* and *F_ST_ = 0.15*.

In each case, the values of 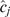 obtained under model 1 and model 2 for each of the eight simulated populations were found very close to the corresponding simulated *F_ST_*: for data set simulated with *F_ST_ = 0.05*, 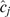 ranged from 0.0437 (0.0442) to 0.0472 (0.0479) under model 1 (model 2) analysis; with *F_ST_ = 0.10*, 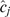 ranged from 0.100 (0.0988) to 0.105 (0.103) under model 1 (model 2) analysis; with *F_ST_ = 0.15*, 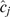 ranged from 0.151 (0.146) to 0.162 (0.151) under model 1 (model 2) analysis. This suggested that estimation of differentiation was more robust to selection than observed above for the pure-drift demographic scenario. As shown in Figure S6, 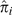 (estimated of reference allele frequency in the gene pool) for neutral SNPs were in good agreement with their corresponding simulated value. Although these estimates remained less precise than previously, they were not sensitive to the level of differentiation since the RRMSE ranged from 16.3% (15.6%) to 16.7% (16.3%) under model 1 (model 2) analysis. As observed previously, the bias was stronger for SNPs subjected to selection but did also not seem dependent on the simulated *F_ST_*. For instance for SNPs subjected to strong positive selection (*s = 0.10*), the RRMSE ranged from 54.8% (60.7%) to 59.9% (67.4%) under model 1 (model 2) analysis. Note that in general, model 2 appeared a little less robust to selection than model 1 when considering estimation of 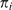.

Consequently, this simple migration drift demographic scenario close to equilibrium appeared more favorable than the previous one to identify SNPs subjected to selection based on the PPP-value criterion (Figure 2). As previously, the mean of the PPP-values was close to 0.5 for both models (from 0.453 to 0.502 with model 1 and from 0.514 to 0.538 with model 2) while standard deviations were lower and decreased with the level of differentiation (from 0.131 to 0.191 with model 1 and from 0.129 to 0.183 with model 2). Likewise, the proportion of SNPs under selection in the tails of the distribution was higher (Table S1). For instance, among the 250 SNPs with the lowest PPP-values, from 92.8% to 98.4% (with model 1) and 100% (with model 2) were subjected to positive selection, while among the 250 SNPs with the highest PPP-values from 29.3% to 45.6% (with model 1) and from 27.3% to 42.6% (with model 2) were under balancing selection. Compared to previous simulation demographic scenarios, model 1 estimates of the PPP-values for SNPs subjected to positive selection deviate to a lesser extent from those estimated with model 2 (Figure 2).

As a consequence, for a given PPP-value threshold, power and robustness to detect SNPs under selection were greatly improved (Table 2). Hence, when looking for SNPs subjected to positive selection, at the 0.2 threshold, FDR was very close to 0 for both models while almost all the SNPs with *s = 0.1* were detected. In general, FDR tended to decrease with differentiation while model 2 performed better than model 1.

### Comparisons with another Bayesian approach on simulated and real data sets

To compare the power and robustness of our decision criterion based on PPP-values to identify SNPs under selection with a previously reported approach, we further analyzed the above simulated data sets under the model initially proposed by Beaumont and Balding [10]. As detailed in the Methods section, this model relies on the estimation, through a logistic regression, of both a “locus effect” and a “population effect” on genetic differentiation. Based on an extension proposed by Riebler *et al.* [13], we recently proposed to derive for each SNP a Bayes Factor (BF) which provides a straightforward decision criterion to decide whether the SNP is subjected to selection [12]. Indeed, in agreement with the Jeffreys' rule [25], we showed that a threshold of 15 (respectively 10) on such BF (expressed in Deciban units) appeared optimal to detect SNPs under positive (respectively balancing) selection. This model and its extensions [10]–[13] might thus be considered as the state of the art Bayesian approach to identify SNPs under selection although it requires far more computational efforts than for models considered in the present study (see Discussion).

**Figure 3:**
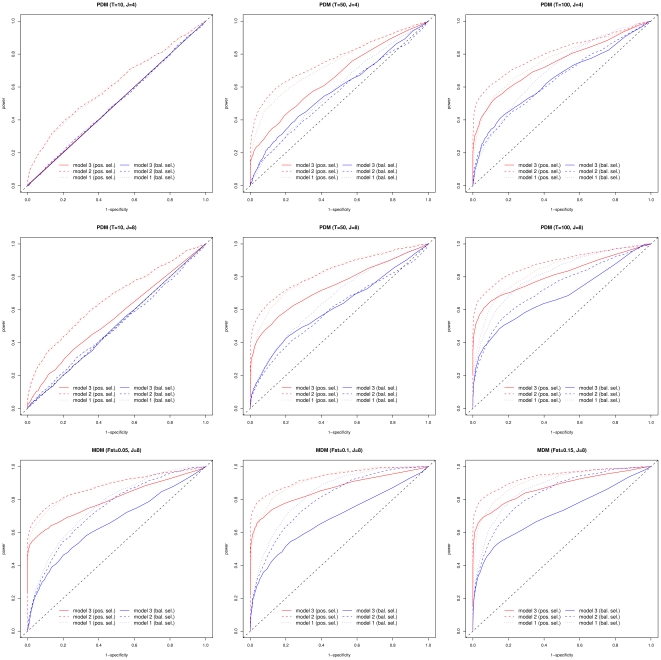
ROC curves for nine simulated data sets obtained with the three different classifiers. Six PDM data sets (*T = 10*, *50* and *100* generations and *J = 4* and *8* populations) and 3 MDM data sets (*F_ST_ = 0.05*, *0.1* and *0.15* and *J = 8* populations) were analyzed. Two ROC curves per analyzed data set were generated (in red for SNPs subjected to positive selection and in blue for SNPs subjected to balancing selection) for each of the three classifiers compared: *i)* classifier based on BF estimated under model 3 (solid line), *ii)* classifier based on PPP-values estimated under model 2 (dashed line) and *iii)* classifier based on PPP-values estimated under model 1 (dotted line).

**Figure 4:**
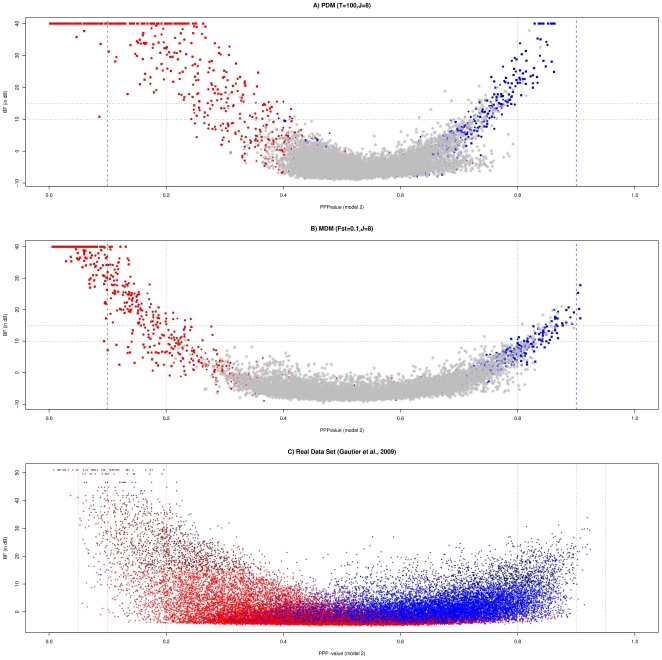
Plots of the PPP-values estimated with model 2 against the BF (in dB) computed with model 3. A) Results for the PDM data set with *T = 100* generations and *J = 8* populations simulated under a pure-drift demographic model. B) Results for the MDM data set with *F_ST_ = 0.1* and *J = 8* populations simulated under a migration-drift demographic model. In A) and B) neutral (simulated) SNPs are plotted in grey while SNPs subjected to positive (respectively balancing) selection are plotted in red (respectively blue). Depending on their underlying simulated coefficient of selection, plots latter are represented by a triangle (*s = 0.02*), a circle (*s = 0.05*) or a square (*s = 0.10*). C) Results from the analysis of the data set consisting in 36,320 SNPs genotyped on 9 West-African cattle populations [12]. SNP localized within the first (last) half of the SNP specific *F_ST_* distribution, as previously estimated [12], are plotted in blue (red), the color being darker for those within the first (last) quarter of this same distribution.

We first assessed the power of the three different classifiers (based on PPP-values estimated under models 1 and 2 and BF estimated under model 3) by generating receiver operating characteristic (ROC) curves [26] which plot the power *vs* (1-specificity) for a binary classification system whereby the cutoff value is varied. Curves resulting from the analysis of nine different simulated data sets are reported in Figure 3 for each of the three classifiers and distinguishing SNPs subjected to positive (in red) and balancing (in blue) selection (irrespectively of the intensities of selection). As expected from previous observations, ROC curves for SNPs subjected to positive selection were always better than ROC curves for SNPs under balancing selection. Similarly, power to detect SNPs subjected to selection under a pure-drift demographic model increased with differentiation but remained lower than under a migration-drift demographic model. Interestingly, ROC curves of both PPP-value classifiers were generally above ROC curves for the BF classifier while the PPP-value classifier based on model 2 clearly outperformed the PPP-value classifier based on model 1 for positive selection. Nevertheless, the definition of an optimal threshold value for the PPP-value classifier strongly depends on the level of differentiation (see above). Table 3 reports the comparisons of the power and robustness of the analyses performed with model 2 and model 3. As a matter of expedience we chose a PPP-value threshold of 0.2 (respectively 0.8) to declare SNPs as subjected to positive (respectively balancing) selection and a threshold of 15 on BF was chosen when analyzing data with model 3. At such thresholds, the FDR were generally similar among the two different analyses except for low level of differentiation (*F_ST_≤0.05*). In these cases FDR (and thus power) was substantially higher for model 2 than for model 3. Note that a great proportion of false positives originated from the upper tail of the PPP-value distribution (see for instance results for the MDM data sets simulated with *F_ST_* = 0.05). At the thresholds considered and for data sets simulated under migration-drift equilibrium, the power to detect SNPs under positive selection varied from 42.9% to 56.8% and was similar between the two approaches. However, for data sets simulated under a pure-drift demographic model, this power was always lower (from 0.3% to 40.9%). In addition, model 3 outperformed model 2 as the level of differentiation increased. Yet, as illustrated in Figure 4A for the PDM data set with T = 100 and J = 8, the PPP-value threshold of 0.2 is clearly too stringent. Hence, for this latter data set, increasing the threshold to 0.3 improves the power from 24.3% to 41.2% (see Table 2), which is similar to the model 3 value (40.9%) without affecting robustness. As expected from previous studies [10]–[13], the power to detect SNPs under balancing selection was small (always lower than 25%). However, model 2 performed generally better than model 3 in particular for MDM data sets.

**Table 3:** Comparison of the power and robustness of models 2 and 3 on simulated data sets.

Simulation Model	FDR (out of 8,500 neutral SNPs)			Power to detect SNP under positive selection			Power to detect SNP under balancing selection		
	model 2 (PPP≤0.2–PPP≥0.8)	model 3 (BF≥15)	models 2 and 3	model 2 (PPP<0.2)	model 3 (BF>15)	models 2 and 3	model 2 (PPP>0.8)	model 3 (BF>15)	models 2 and 3
PDM (T = 10, J = 4)	17.5% (8.9%–8.5%)	-	-	22/46/108 (23.5%)	-	-	18/21/24 (8.4%)	-	-
PDM (T = 25, J = 4)	1.5% (0.3%–1.2%)	-	-	2/17/104 (16.4%)	0/0/8 (1.1%)	0/0/8 (1.1%)	3/3/8 (1.9%)	-	-
PDM (T = 50, J = 4)	-	-	-	0/3/84 (11.6%)	0/1/43 (5.9%)	0/1/36 (4.9%)	-	-	-
PDM (T = 75, J = 4)	-	-	-	0/7/99 (14.1%)	0/6/90 (12.8%)	0/3/71 (9.9%)	-	-	-
PDM (T = 100, J = 4)	-	-	-	0/11/88 (13.2%)	0/12/122 (17.9%)	0/6/80 (11.5%)	-	-	-
PDM (T = 10, J = 8)	21.8% (9.4%–12.3%)	-	-	16/68/150 (31.2%)	0/0/2 (0.3%)	0/0/2 (0.3%)	29/37/35 (13.5%)	-	-
PDM (T = 25, J = 8)	4.5% (0.3%–4.1%)	-	-	5/32/147 (24.5%)	0/0/65 (8.7%)	0/0/65 (8.7%)	8/18/29 (7.3%)	-	-
PDM (T = 50, J = 8)	0.6% (0.0%–0.6%)	0.1%	0.1%	0/24/132 (20.8%)	0/15/141 (20.8%)	0/11/115 (16.8%)	3/12/33 (6.4%)	0/1/5 (0.8%)	0/1/3 (0.5%)
PDM (T = 75, J = 8)	0.2% (0.0%–0.2%)	0.3%	0.2%	0/16/171 (24.9%)	0/48/223 (36.1%)	0/15/168 (24.4%)	1/15/35 (6.8%)	0/18/32 (6.7%)	0/15/29 (5.9%)
PDM (T = 100, J = 8)	0.1% (0.0%–0.1%)	0.3%	0.1%	0/20/162 (24.3%)	1/72/234 (40.9%)	0/20/161 (24.1%)	0/8/35 (5.7%)	3/22/63 (11.7%)	0/8/35 (5.7%)
MDM (F_ST_ = 0.05, J = 8)	5.6% (0.2%–5.4%)	-	-	11/169/246 (56.8%)	0/99/223 (42.9%)	0/99/223 (42.9%)	26/67/94 (24.9%)	0/2/1 (0.4%)	0/2/1 (0.4%)
MDM (F_ST_ = 0.1, J = 8)	0.9% (0.0%–0.9%)	0.1%	0.1%	1/146/242 (51.9%)	2/128/219 (46.5%)	1/124/219 (45.9%)	13/29/73 (15.3%)	1/4/15 (2.7%)	1/4/15 (2.7%)
MDM (F_ST_ = 0.15, J = 8)	0.1% (0.0%–0.1%)	0.1%	0.1%	1/114/223 (45.1%)	2/111/215 (43.7%)	1/102/210 (41.7%)	6/22/58 (11.5%)	2/15/39 (7.5%)	2/15/38 (7.3%)

For model 2, a PPP-value threshold of 0.2 (respectively 0.8) was chosen to declare SNPs as being under positive (respectively balancing) selection. For model 3, a threshold of 15 on *BF* (expressed in dB units) was chosen. The table reports, for each simulated data set and analysis, the percentage of neutral SNPs (among the 8,500 simulated ones) which are incorrectly declared as under selection (with model 2, model 3 and in both models 2 and 3 analyses). In addition, for each type of selection (positive or balancing), the numbers of SNPs under selection among the 250 simulated for each of the three classes of selection coefficient (*s = 0.02*/*s = 0.05*/*s = 0.10*) which are correctly identified is also reported. The global power (out of the 750 SNPs simulated for each type of selection) is given in parentheses.

Finally, as shown in Table 3 and Figure 4, results were in good agreement between the two models since a good relationship appeared between estimated PPP-values and BF (the more extreme PPP-value towards 0 or 1, the higher the BF). As an example, in the MDM simulated data set with *F_ST_* = 0.15, respectively 338 and 328 SNPs were (correctly) identified as under positive selection with analysis based on model 2 and model 3 (Table 3). Among these, 313 SNPs were shared by both procedures.

### Analysis of a cattle data set

We finally analyzed a data set consisting of 9 West-African cattle populations genotyped for 36,320 SNPs we previously used to perform of whole genome scan for adaptive divergence [12]. Overall, estimates of population specific differentiation were in good agreement with those previously reported (Table S2) especially, and as expected, when using model 2. Nevertheless, for highly differentiated populations (*F_ST_*>0.2), model 1 resulted in higher values. The *F_ST_* averaged across all populations ranged from 0.141 to 0.160, depending on the model considered. In addition, in our initial analysis [12] we also derived estimates for SNP specific *F_ST_*. Note that these latter were computed as the median of the corresponding posterior distributions (thus leading, when averaging across SNPs, to a lower global *F_ST_* of 0.100).

We further studied the distribution of PPP-values estimated for each SNPs. As with simulated data, the average PPP-value was close to 0.5 with both models (0.480 with model 1 and 0.521 with model 2) with an almost equal standard deviation (0.173 with model 1 and 0.172 with model 2). When comparing these results to those previously obtained (Figure 4C), an overall good agreement was observed. Indeed, the higher the SNPs were differentiated (plotted in blue in the Figure 4C) and the higher the BF, the lower the estimated PPP-values. Conversely, the lower the SNPs were differentiated (plotted in red in the Figure 4C) and the higher the BF, the higher the estimated PPP-values. However, although the estimates from the two models lead to qualitatively similar results with a global correlation of 0.928 between them, the dispersion was higher for those obtained with model 2 (from 0.008 to 0.924) than model 1 (from 0.012 to 0.902). In particular, in the tails of the distributions PPP-values were generally more extreme with model 2 than model 1. This observation was actually in good agreement with results obtained on simulated data sets (see above). As a consequence, model 2 lead to a decision regarding SNP selection status more in agreement with the one based on the BF [12]. For instance, we initially identified 2,054 SNPs with a BF>15 as candidates to be subjected to selection, among which 537 were most likely under balancing selection (*F_ST_*<0.011) while 1,517 were most likely under positive selection (*F_ST_*>0.28). For the first set (537 SNPs), PPP-values ranged from 0.363 to 0.902 (0.775 on average) when considering model 1 results and from 0.339 to 0.924 (0.781 on average) with model 2. For the second set (1,517 SNPs), PPP-values ranged from 0.0120 to 0.604 (0.262 on average) when considering model 1 results and from 0.008 to 0.407 (0.200 on average) with model 2.

## Discussion

In this study, we implement a Bayesian model-based strategy to scan for adaptive differentiation on large SNP data sets. The rationale of such approaches consists of evaluating a local adjustment of the null model to data at each locus by computing PPP-values [15]. We then investigated two different models (model 1 and model 2), which could respectively be interpreted in the context of pure-drift divergence [17] and an infinite Wright island model involving drift and migration at its equilibrium state [10], [18]. An important feature of both models was the possibility to derive population-specific differentiation estimates [18]. These latter parameters are then expected to be constant over SNPs within each population under the neutral hypothesis corresponding to SNP exchangeability. Consequently, SNPs displaying either high or low PPP-values (*i.e.* outliers) were interpreted as loci possibly under selection. From a theoretical point of view, such likelihood-based strategy permits full use of the data, relying explicitly on a model rather than opting for the identification of outliers based on an empirical or simulated distribution of summary statistics [7]–[9]. Conversely, in our approach, the integration over the posterior distribution of parameters, given the complete data vector in the calculation of PPP-values, results in a rather conservative procedure (double use of the data). However, and as pointed out by Bayarri and Castellanos [27], a small (or large) PPP-value “can safely be interpreted as incompatibility with the null model”. Nevertheless, compressing the data into summary statistics and further replacing likelihood computation by data simulations under an Approximated Bayesian Computation (ABC) framework was recently proven efficient to identify loci subjected to selection while allowing complex genetic model to be considered [28].

Evaluation of both models on simulated data sets revealed that they performed equally in estimating differentiation when considering a migration-drift scenario while model 2 was surprisingly more efficient under the pure-drift divergence scenario. For the latter scenario, nevertheless, they together resulted in underestimation of differentiation at low level of pure-drift divergence (*F_ST_*<0.05) while model 1 implementation became fairly inaccurate (*e.g.* Figure S2A) for high level of divergence (*F_ST_*>0.2). Actually, although the *Beta* distribution (with parameters depending on ancestral or gene pool allele frequency and differentiation) does not consider the allele fixation, this distribution provided a better fit to the one expected in the pure-drift case [24] as depicted in Figure S5. Thus, even though model 1 took into account the possibility of allele fixation by introducing a truncated Gaussian distribution, it did not provide any gain in estimation robustness.

As expected from previous studies [10]–[13], estimated PPP-values allowed more accurate identification of SNPs under positive rather than balancing selection. Moreover, the accuracy increased with the intensity of selection. Importantly, the dispersion of PPP-values was confirmed to be highly dependent on the level of differentiation since this is directly related to the variance in allele frequency. In particular, for the case of low level of pure-drift divergence (*F_ST_*<0.05) for which we observed an underestimation of differentiation, this might contribute to an increased FDR. More generally and from a desired practical point of view, this made it difficult to propose standard thresholds on the distribution of PPP-values to decide whether or not a SNP is under selection, although more sophisticated approaches could have been envisioned such as clustering techniques based on mixture models. However, at this stage, we wanted to keep the decision rule as simple as possible. Thus, as a matter of expedience, to detect SNP candidates to be subjected to positive (balancing) selection, a conservative threshold of 0.1 (0.9) might be recommended since in most cases investigated through simulation it lead to a FDR close to 0 (although it increases when differentiation decreases) and an optimal power (which conversely decreases as differentiation increases). When analyzing the two types of simulated data sets, it clearly appeared that both models were less powerful when dealing with completely isolated populations. However and more strikingly, model 2 undoubtedly outperformed model 1 in identifying outliers probably because it resulted in more extreme PPP-value estimates for SNPs localized in either tail of the distribution. This trend was confirmed when comparing results with those based on the alternative and more complex modeling represented by model 3 [10]–[13]. More generally, both approaches were shown to have similar performances. This might be related to the high similarity between the two underlying statistical models. Note however that while the PPP-value is essentially a measure of the departure from the null hypothesis (SNP neutrality), under the approach based on model 3, an explicit modeling of the alternative hypothesis is performed through the introduction of a SNP effect in a logistic regression of the differentiation.

For real data sets, the SNP ascertainment process might also be expected to affect robustness of the approaches. Hence, approaches to perform a separate modeling of the demographic and ascertainment processes have recently been proposed [29]. However, as previously discussed [12] and suggested by our simulations, the different models appeared rather robust to such a bias. This might result from their insensitiveness to the prior distribution on the 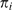. As a consequence, the main difficulties might be more related to the simple assumptions on which demographic scenarios relied. Except for some special cases (*e.g.* artificial selection experiments based on the development of divergent lines from a common founder population), assuming star shaped phylogenies, as in a pure-drift model, remains highly unrealistic and leads to an underestimation of the variance in allele frequency when a hierarchical structure exists among the populations studied [30]. Due to the dependency of PPP-values on this crucial parameter, we might have expected a higher rate of false positives in these situations. For instance, it was recently shown that such population relationships greatly increased the rate of false positives in tests of selection based on *F_ST_* which use a null distribution generated under a simple island model of differentiation [31]. Nevertheless, simulations under more complex scenarios suggested that the approach was relatively robust to departure from simple demographic assumptions provided the level of differentiation was not too low. Interestingly, hierarchical structure among populations as introduced from recent admixture or population splitting appeared as limiting cases. Indeed, we observed a high underestimation of the population specific differentiation parameter for recently admixed (or split) populations, leading to an increase of the PPP-values dispersion. Owing to the flexibility of Bayesian hierarchical modeling, it might be straightforward to include additional levels in models 1 and 2 to incorporate prior information on relationships among the populations surveyed. Alternatively, in the context of high throughput genotyping data sets, results from different neighbor SNPs might be empirically combined to identify regions in the genome displaying an unexpectedly high proportion of outliers [12]. Such regional information was overlooked in both our models since we considered all SNPs as exchangeable. Introduction of a spatial structure among SNPs was recently investigated, to take Linkage Disequilibrium into account in a model extending model 2 [14]. However, when considered on a whole genome basis, such approaches might add significant computational burden.

Finally, the model-based strategy presented in this study was chiefly operational and this might be viewed as an efficient way to perform a first exploratory analysis of large data sets. Hence running the MCMC algorithm underlying model 1 for 250,000 iterations on a data set containing 10,000 SNPs genotyped on 10 populations needed approximately 2 hours on a PC equipped with a 2.1 GHz processor. In contrast, the analysis of the cattle data set with model 3 [10], [12], [13] took more than 40 hours (*i.e.* approximately 20 times slower).

## Supporting Information

Text S1 Description of the MCMC algorithms.
(0.09 MB PDF)

Text S2 Derivation of E(f_ij_|θ_ij_) and Var(f_ij_|θ_ij_).
(0.01 MB PDF)

Text S3 Simulation study design.
(0.03 MB PDF)

Table S1 Composition of the tails of the PPP-values distribution. Results for eight different data sets are reported, five of which were simulated under a pure drift demographic model (PDM) with a varying number T of simulated generations and three were simulated under a migration drift model (MDM) with a varying level of migration controlled by the simulated FST. For each simulated data sets and analysis (either with model 1 or model 2), the proportions of SNPs subjected to selection within the considered tail of the distribution which belong to the different classes of selection type (N = neutral, B = balancing or P = positive) and selection coefficients (s = 0.02, s = 0.05 and s = 0.10) are reported.
(0.04 MB XLS)

Table S2 Estimates of differentiation for the nine populations from the cattle data set obtained with model 1, model 2 and the alternative model used in the initial report [12].
(0.01 MB XLS)

Figure S1 95% equal tail Credible Interval for the differentiation parameter c obtained after analyzing two data sets DS1 and DS2 simulated respectively under inference model 1 and 2. The two simulated data sets consist of 1,000 SNPs and 12 populations with the following simulated value of c : c1 = c2 = 0.01, c3 = c4 = 0.025, c5 = c6 = 0.05, c7 = c8 = 0.075, c9 = c10 = 0.1 and c11 = c12 = 0.15. A) Data set DS1 analyzed with model 1, B) Data set DS1 analyzed with model 2, C) Data set DS2 analyzed with model 2, D) Data set DS2 analyzed with model 2.
(0.03 MB PDF)

Figure S2 Estimates of c for 17 data sets simulated under a pure-drift demographic model. Allele counts for 1,000 (neutral) SNPs were simulated for 10 populations and for 23 different times (measured in number of discrete generations) after divergence (T = 10, T = 20, T = 30, T = 40, T = 50, T = 60, T = 70, T = 80, T = 90, T = 100, T = 125, T = 150, T = 175, T = 200, T = 250, T = 300, T = 400, T = 500, T = 600, T = 700, T = 800, T = 900 and T = 1000). The resulting data sets were analyzed using both model 1 (A) and model 2 (B). Resulting estimates (mean of the posterior distribution) are plotted against the corresponding simulated time (the different number representing the population label) and are connected by a line. The grey dashed line represents the expected FST value (see Methods).
(0.07 MB PDF)

Figure S3 Allele frequency distribution within a population of constant (haploid) effective size (Ne = 500) evolving during T discrete generations (T = log(1−c)/log(1−1/Ne) where c is a measure a population differentiation) as a function of the initial allele frequency (Pi). For each case investigated, a histogram of 1,000,000 simulated values is plotted together with the corresponding densities from model 1 (truncated Gaussian in blue with probability masses in 0 and 1) and model 2 (Beta distribution). Note that an exact derivation of the corresponding distribution has been derived using a forward-time diffusion approach [24].
(0.32 MB PDF)

Figure S4 Estimates of c for five data sets simulated under a pure-drift demographic model. Allele counts for 10,000 SNPs (8,500 neutral SNPs, 750 subjected to positive selection and 750 to balancing selection) were simulated for 8 populations and for 5 different times (measured in number of discrete generations) after divergence (T = 10, T = 25, T = 50, T = 75 and T = 100). The five resulting data sets were analyzed using both model 1 (A) and model 2 (B). Resulting estimates (mean of the posterior distribution) are plotted against the corresponding simulated time (the different number representing the population label) and are connected by a line. The grey dashed line represents the expected FST value (see Methods).
(0.01 MB PDF)

Figure S5 Robustness of the estimates (mean of the posterior distribution) of the ancestral (reference) allele frequency πi. Two data sets (T = 10 and T = 100) consisting in genotyping data for 10,000 SNPs (8,500 neutral SNPs, 750 subjected to positive selection and 750 subjected to balancing selection) on 8 populations were analyzed with model 1 and model 2 (see text). For each data set, three plots are shown: i) estimates obtained under model 1 against (true) simulated values (A with T = 10 and D with T = 100), ii) estimates obtained under model 2 against (true) simulated values (B with T = 10 and E with T = 100) and iii) estimates obtained under model 1 against estimates obtained under model 2 (C with T = 10 and E with T = 100). Neutral SNPs are plotted in grey while SNPs subjected to positive (respectively balancing) selection are plotted in red (respectively blue). In addition, the simulated coefficients of selection are represented by a triangle (s = 0.02), a circle (s = 0.05) or a square (s = 0.10).
(1.06 MB JPG)

Figure S6 Robustness of the estimates (mean of the posterior distribution) of the (reference) allele frequency πi in the gene pool. Two data sets (FST = 0.05 and FST = 0.15) consisting in genotyping data for 10,000 SNPs (8,500 neutral SNPs, 750 subjected to positive selection and 750 subjected to balancing selection) on 8 populations were analyzed with model 1 and model 2 (see text). For each data set, three plots are represented: i) estimates obtained under model 1 against (true) simulated values (A with FST = 0.05 and D with FST = 0.15), ii) estimates obtained under model 2 against (true) simulated values (B with FST = 0.05 and E with FST = 0.15) and iii) estimates obtained under model 1 against estimates obtained under model 2 (C with FST = 0.05 and E with FST = 0.15). Neutral SNPs are plotted in grey while SNPs subjected to positive (respectively balancing) selection are plotted in red (respectively blue). In addition, the simulated coefficients of selection are represented by a triangle (s = 0.02), a circle (s = 0.05) or a square (s = 0.10).
(1.51 MB JPG)

## References

[1] Beaumont M, Nichols RA (1996). Evaluating loci for use in the genetic analysis of population structure.. Proc R Soc Lond B.

[2] Lewontin RC, Krakauer J (1973). Distribution of gene frequency as a test of the theory of the selective neutrality of polymorphisms.. Genetics.

[3] Nielsen R (2005). Molecular signatures of natural selection.. Annu Rev Genet.

[4] Storz JF (2005). Using genome scans of DNA polymorphism to infer adaptive population divergence.. Mol Ecol.

[5] Vitalis R, Dawson K, Boursot P (2001). Interpretation of variation across marker loci as evidence of selection.. Genetics.

[6] Bowcock AM, Kidd JR, Mountain JL, Hebert JM, Carotenuto L (1991). Drift, admixture, and selection in human evolution: a study with DNA polymorphisms.. Proc Natl Acad Sci U S A.

[7] Akey JM, Zhang G, Zhang K, Jin L, Shriver MD (2002). Interrogating a high-density SNP map for signatures of natural selection.. Genome Res.

[8] Flori L, Fritz S, Jaffrezic F, Boussaha M, Gut I (2009). The genome response to artificial selection: a case study in dairy cattle.. PLoS One.

[9] Weir BS, Cardon LR, Anderson AD, Nielsen DM, Hill WG (2005). Measures of human population structure show heterogeneity among genomic regions.. Genome Res.

[10] Beaumont MA, Balding DJ (2004). Identifying adaptive genetic divergence among populations from genome scans.. Mol Ecol.

[11] Foll M, Gaggiotti O (2008). A genome-scan method to identify selected Loci appropriate for both dominant and codominant markers: a bayesian perspective.. Genetics.

[12] Gautier M, Flori L, Riebler A, Jaffrezic F, Laloe D (2009). A whole genome Bayesian scan for adaptive genetic divergence in West African cattle.. BMC Genomics.

[13] Riebler A, Held L, Stephan W (2008). Bayesian variable selection for detecting adaptive genomic differences among populations.. Genetics.

[14] Guo F, Dey DK, Holsinger KE (2009). A Bayesian hierarchical model for analysis of SNP diversity in multilocus, multipopulation samples.. J Am Stat Assoc.

[15] Gelman A, Meng XL, Stern H (1996). Posterior Predictive Assessment of Model Fitness via Realized Discrepancies.. Statistica sinica.

[16] Rubin DB (1984). Bayesianly justifiable and relevant frequency calculations for the applied statistician.. Ann Statist.

[17] Nicholson G, Smith AV, Jonsson F, Gustafsson O, Stefansson K (2002). Assessing population differentiation and isolation from single-nucleotide polymorphism data.. Journal of the Royal Statistical Society: Series B (Statistical Methodology).

[18] Balding DJ (2003). Likelihood-based inference for genetic correlation coefficients.. Theor Popul Biol.

[19] Foll M, Beaumont MA, Gaggiotti O (2008). An approximate Bayesian computation approach to overcome biases that arise when using amplified fragment length polymorphism markers to study population structure.. Genetics.

[20] Thomas A, O Hara B, Ligges U, Sturtz S (2006). Making BUGS Open.. R News.

[21] Gelman A, Carlin JB, Stern HS, Rubin DB (2004). Bayesian Data Analysis, 2nd edition.

[22] Schaffner SF, Foo C, Gabriel S, Reich D, Daly MJ (2005). Calibrating a coalescent simulation of human genome sequence variation.. Genome Res.

[23] Kong A, Gudbjartsson DF, Sainz J, Jonsdottir GM, Gudjonsson SA (2002). A high-resolution recombination map of the human genome.. Nat Genet.

[24] Crow JF, Kimura M (1970). An Introduction to Population Genetics Theory.

[25] Jeffreys H (1961). Theory of Probability, 3rd edition.

[26] Fawcett T (2006). An introduction to ROC analysis.. Pattern Recogn Lett.

[27] Bayarri MJ, Castellanos ME (2007). Bayesian Checking of the Second Level of Hierarchical Models (Discussion Paper).. Statistical Science.

[28] Bazin E, Dawson KJ, Beaumont MA (2010). Likelihood-free Inference of Population Structure and Local Adaptation in a Bayesian Hierarchical Model.. Genetics.

[29] Guillot G, Foll M (2009). Correcting for ascertainment bias in the inference of population structure.. Bioinformatics.

[30] Robertson A (1975). Gene frequency distributions as a test of selective neutrality.. Genetics.

[31] Excoffier L, Hofer T, Foll M (2009). Detecting loci under selection in a hierarchically structured population.. Heredity.

