# Supplementary material for: A Bayesian Outlier Criterion to Detect SNPs under Selection in Large Data Sets
Source: PLoS One. 2010 Aug 2;5(8):e11913. doi: 10.1371/journal.pone.0011913 (PMC2914027; doi:10.1371/journal.pone.0011913)
Supplement: Text S1 — Description of the MCMC algorithms. (0.09 MB PDF) [file pone.0011913.s001.pdf]

A Metropolis-Hastings (MH) algorithm within Gibbs was implemented to generate the MCMC chains used to estimate the posterior distribution of each parameter of interest. At each iteration t of the algorithm, the parameters α (or β), π and c (ignoring the subscripts *i* and *j* for the sake of simplicity) are sampled according to the following steps.

1) Letting θ_k the kth parameter of interest and θ_k^t its current value at iteration t, generate a candidate $\tilde{\theta}_k^{t+1}$ according to a uniform distribution

$$\tilde{\theta}_k^{t+1} \sim \mathrm{U}\left(\min(0,\theta_k^t - \delta_k / 2), \max(1, \theta_k^t + \delta_k / 2)\right) \qquad \text{(I.1)}$$

where δ_k is the range of variation allowed for the random walk in the uniform distribution around θ_k^t.

2) Compute

$$\rho_k^{t+1} = \frac{p\left(\tilde{\theta}_k^{t+1} \mid \theta_{[k]}^c, x\right) \times \left(\max(1, \tilde{\theta}_k^{t+1} + \delta_k / 2) - \min(0, \tilde{\theta}_k^{t+1} - \delta_k / 2)\right)}{p\left(\theta_k^t \mid \theta_{[k]}^c, x\right) \times \left(\max(1, \theta_k^t + \delta_k / 2) - \min(0, \theta_k^t - \delta_k / 2)\right)}, \text{(I.2)}$$

where ρ_k^{t+1} is the ratio of the densities of the conditional distribution of the candidate $\tilde{\theta}_k^{t+1}$ and of the current value θ_k^t (given the data x and the other parameters $\theta_{[k]} = \theta \setminus \theta_k$ being equal to their current values $\theta_{[k]}^c$) times the ratio of the densities of the proposal for the current and the candidate values.

3) Generate $u_k^{t+1} \sim U\left(0,1\right)$, a random uniform number on $\left(0,1\right)$

4) If $u_k^{t+1} \le \rho_k^{t+1}$, then take $\theta_k^{t+1} = \tilde{\theta}_k^{t+1}$, otherwise $\theta_k^{t+1} = \theta_k^t$.

The range of variation δ_k for the different proposal distributions were initially adjusted during several successive pilot runs (*e.g.* of 1000 iterations) until achieving an acceptance rate for each parameters ranging from 0.25 to 0.4.

Now, we have to specify the density $p\left(.|\theta_{[k]}^{c},x\right)$ involved in the computation of ρ_k^{t+1} for each parameter in models 1 and 2.

In model 1, we have

i) $$p(\beta|\pi,c,x) \propto p(\beta|\pi,c)\,p(x|\beta,\pi,c) \qquad \text{(I.3)}$$

with $\beta|\pi,c \sim N\left[\pi,c\pi(1-\pi)\right]$ and $x|\beta,\pi,c \equiv x|\beta \sim B(n,\alpha)$;

ii) $$p(\pi|\beta,c,x) \equiv p(\pi|\beta,c) \propto p(\beta|\pi,c)\,p(\pi). \qquad \text{(I.4)}$$

iii) $$p(c|\beta,\pi,x) \equiv p(c|\beta,\pi) \propto p(\beta|\pi,c)\,p(c). \qquad \text{(I.5)}$$

Notice that in the last two cases ii) and iii) the conditional densities no longer depend of the data.

The same scheme applies to model 2 except that sampling in the conditional $\alpha|\pi,x$ is carried out directly due to the conjugacy property of the beta-binomial model so that

$$\alpha|\pi,x \sim Beta\left[\tau\pi+x,\tau(1-\pi)+n-x\right]. \qquad \text{(I.6)}$$

Typical runs of these two MCMC algorithms consisted generally in few tens of pilot runs, followed by 10,000 burn-in iterations. The posterior distributions were then computed from 5,000-10,000 post burn-in iterations with a thinning of 50 iterations (leading to 250,000-500,000 post burn-in iterations). Convergence of the MCMC was checked using standard criteria.
