# Supplementary material for: A Bayesian Outlier Criterion to Detect SNPs under Selection in Large Data Sets
Source: PLoS One. 2010 Aug 2;5(8):e11913. doi: 10.1371/journal.pone.0011913 (PMC2914027; doi:10.1371/journal.pone.0011913)
Supplement: Text S2 — Derivation of E(fij|θij) and Var(fij|θij). (0.01 MB PDF) [file pone.0011913.s002.pdf]

We can express the binomial random variable x_{ij} as the sum $x_{ij} = \sum_{k=1}^{n_{ij}} x_{ijk}$ of independent and identically distributed Bernoulli variable $x_{ijk} \sim Ber\left(\alpha_{ij}\right)$. We have to derive the expression of the first two moments of x_{ij}. We have

$$E\left(x_{ij}\right) = n_{ij} E\left(x_{ijk}\right) \qquad \text{(II.1)}$$

and

$$Var\left(x_{ij}\right) = \sum_k Var\left(x_{ijk}\right) + 2\sum_{k<k'} Cov\left(x_{ijk}, x_{ijk'}\right) \qquad \text{(II.2)}$$

Using the conditioning-deconditioning theorem,

$$E\left(x_{ijk}\right) = E_{\alpha_{ij}}\left(x_{ijk} \mid \alpha_{ij}\right) = E\left(\alpha_{ij}\right) \qquad \text{(II.3)}$$

$$Var\left(x_{ijk}\right) = E_{\alpha_{ij}}\left[Var\left(x_{ijk} \mid \alpha_{ij}\right)\right] + Var_{\alpha_{ij}}\left[E\left(x_{ijk} \mid \alpha_{ij}\right)\right]$$

Now, $Var\left(x_{ijk} \mid \alpha_{ij}\right) = \alpha_{ij}\left(1-\alpha_{ij}\right)$ so that

$$Var\left(x_{ijk}\right) = E\left(\alpha_{ij}\right) - E\left(\alpha_{ij}^2\right) + Var\left(\alpha_{ij}\right) \qquad \text{(II.4)}$$

Similarly

$$Cov\left(x_{ijk}, x_{ijk'}\right) = E_{\alpha_{ij}}\left[Cov\left(x_{ijk}, x_{ijk'} \mid \alpha_{ij}\right)\right] + Cov_{\alpha_{ij}}\left[E\left(x_{ijk} \mid \alpha_{ij}\right), E\left(x_{ijk'} \mid \alpha_{ij}\right)\right].$$

x_{ijk} and $x_{ijk'}$ being conditionally independent, this expression reduces to

$$Cov\left(x_{ijk}, x_{ijk'}\right) = Var\left(\alpha_{ij}\right) \qquad \text{(II.5)}$$

Moreover

$$E\left(\alpha_{ij}\right) = \pi_i,\ Var\left(\alpha_{ij}\right) = c_j \pi_i \left(1-\pi_i\right). \qquad \text{(II.6)}$$

These expressions are exact under model 2 and approximate under model 1 due to the truncation. Then substituting (II.6) into (II.4), (II.5) and (II.1), (II.2) gives

$$E\left(x_{ij}\right)=n_{ij}\pi_i$$

$$Var\left(x_{ij}\right)=n_{ij}\pi_i\left(1-\pi_i\right)\left[1+\left(n_{ij}-1\right)c_j\right]$$

from which we can immediately deduce the homologous expressions for the frequency $f_{ij}=x_{ij}/n_{ij}$.
