# Supplementary material for: A Bayesian Outlier Criterion to Detect SNPs under Selection in Large Data Sets
Source: PLoS One. 2010 Aug 2;5(8):e11913. doi: 10.1371/journal.pone.0011913 (PMC2914027; doi:10.1371/journal.pone.0011913)
Supplement: Text S3 — Simulation study design. (0.03 MB PDF) [file pone.0011913.s003.pdf]

1) Simulation under a pure-drift demographic model with selection

Let $y_{ij,t}$ be the (reference) allele count for SNP i $(1 \le i \le L)$ in population j $(1 \le j \le P)$ at time t $(0 \le t \le T)$. Similarly, let π_i be the (reference) allele frequency in the ancestral population and N_j the (constant) diploid size of population j. At generation $t = 0$, the π_i's were sampled from beta distributions with parameters 0.7 and 0.7 and truncated at 0.05 and 0.95 (to avoid a surplus of very low and high frequencies).

The corresponding $y_{ij(0)}$ were then sampled from a binomial distribution $y_{ij,0} \sim B(2N_j, \pi_i)$. Selection is further introduced in the model by attaching a selective coefficient s_i ($s_i = 0$ for a neutral locus) and a selection type (either positive or balancing) to each SNP. The simulation model for a particular SNP i within a particular population j proceeds as follows:

1. Following Beaumont and Balding [10], assign randomly the attribute (blue, b; red, r; or neutral, n) for population j at SNP i with $p_{b,j} = 0.4$, $p_{r,j} = 0.4$ and $p_{n,j} = 0.2$

2. To model genetic drift, sample $y^*_{ij,t}$ from a binomial distribution $y^*_{ij,t} \sim B(2N_j, y_{ij,t-1}/2N_j)$

3 To model selection, update the observed allele frequency $p^*_{ij,t} = y^*_{ij,t}/2N_j$ as follows:

31 Determine the allele fitness $w_{ij}^{(.)}$ of each possible genotype assuming Hardy-Weinberg equilibrium. If the SNP is subjected to positive selection $w_{ij}^{11} = 1 + \delta_{ij} s_i$ (homozygous for the reference allele) , $w_{ij}^{12} = 1 + ½ s_i$ (heterozygous) and $w_{ij}^{22} = 1 + (1 - \delta_{ij}) s_i$ (homozygous for the alternative allele) where $\delta_{ij} = 1$ (respectively $\delta_{ij} = 0$) if the attribute of population j for SNP i is “blue” (respectively “red”) and

$w_{ij}^{11} = w_{ij}^{12} = w_{ij}^{22} = 1$ if the attribute of population j for SNP i is "neutral". If the SNP is subjected to balancing selection $w_{ij}^{11} = w_{ij}^{22} = 1$; and $w_{ij}^{12} = 1 + s_i$ if the attribute of population j for SNP i is "blue" or "red" and $w_{ij}^{12} = 1$ if it is "neutral".

32 Compute the mean fitness $\overline{w}_{ij,t}$ for population j assuming Hardy–Weinberg equilibrium and calculate the allele count $y_{ij,t}^{*}$ for the next generation from

$$\overline{w}_{ij,t} = p_{ij,t}^{*2} w_{ij}^{(11)} + 2 p_{ij,t}^{*} \left(1 - p_{ij,t}^{*}\right) w_{ij}^{(12)} + \left(1 - p_{ij,t}^{*}\right)^2 w_{ij}^{(22)}$$

and

$$y_{ij,t}^{*} = 2N_j \left[p_{ij,t}^{*2} w_{ij}^{(11)} + p_{ij,t}^{*} \left(1 - p_{ij,t}^{*}\right) w_{ij}^{(12)} \right] / \overline{w}_{ij,t}$$

4 Go to step 2 until $t=T$

2) Simulation under a migration-drift demographic model (at equilibrium) with selection

Migration Drift model with selection was simulated according to a simplified version of previously proposed algorithms [10,13]. The simulation model was thus similar to the one describe above in AIII1 except that an additional population parameter *F* was considered to allow populations to exchange migrant alleles through a common gene pool. Thus, π_i now represented the constant (reference) allele frequency in this gene pool. A step simulating migration was then further introduced between step 2 (genetic drift) and step 3 (selection) as follows:

1) Compute the immigration rate as $m_j = (1 - F) / 4N_j F$

2) Sample the number of immigrants (chromosomes) n_j^{im} entering population j as $n_j^{im} \sim B\left(2N_j, m_j\right)$

3) Generate the number of immigrants n_{-j}^{im} chromosomes from each population (other than j) as $\mathbf{n}_{-j}^{im} \sim M\left(n_j^{im}, \mathbf{p}_{-j}^{m}\right)$ where $\mathbf{p}_{-j}^{m}$ is a vector with $P-1$ elements all equal to $1/(P-1)$

4) Determine the number of reference alleles n_1^{gain} among the immigrants by sampling a binomially-distributed number from each population other than j (with parameters equal to the corresponding number of immigrants chromosomes and reference allele frequencies)

5) Determine the number of chromosomes n_1^{lost} lost which carry the reference allele among the $n_j^{mig} = n_j^{im}$ migrants as $n_1^{lost} \sim B\left(n_j^{mig}, p_{ij,t}^{*}\right)$

6) Update allele frequency as $p_{ij,t}^{*} = p_{ij,t}^{*} + \dfrac{n_1^{gain} - n_1^{lost}}{2N_j}$
