# Supplementary figures and images for: A Bayesian Outlier Criterion to Detect SNPs under Selection in Large Data Sets
Source: PLoS One. 2010 Aug 2;5(8):e11913. doi: 10.1371/journal.pone.0011913 (PMC2914027; doi:10.1371/journal.pone.0011913)

**A) DS1 analyzed with Model 1**

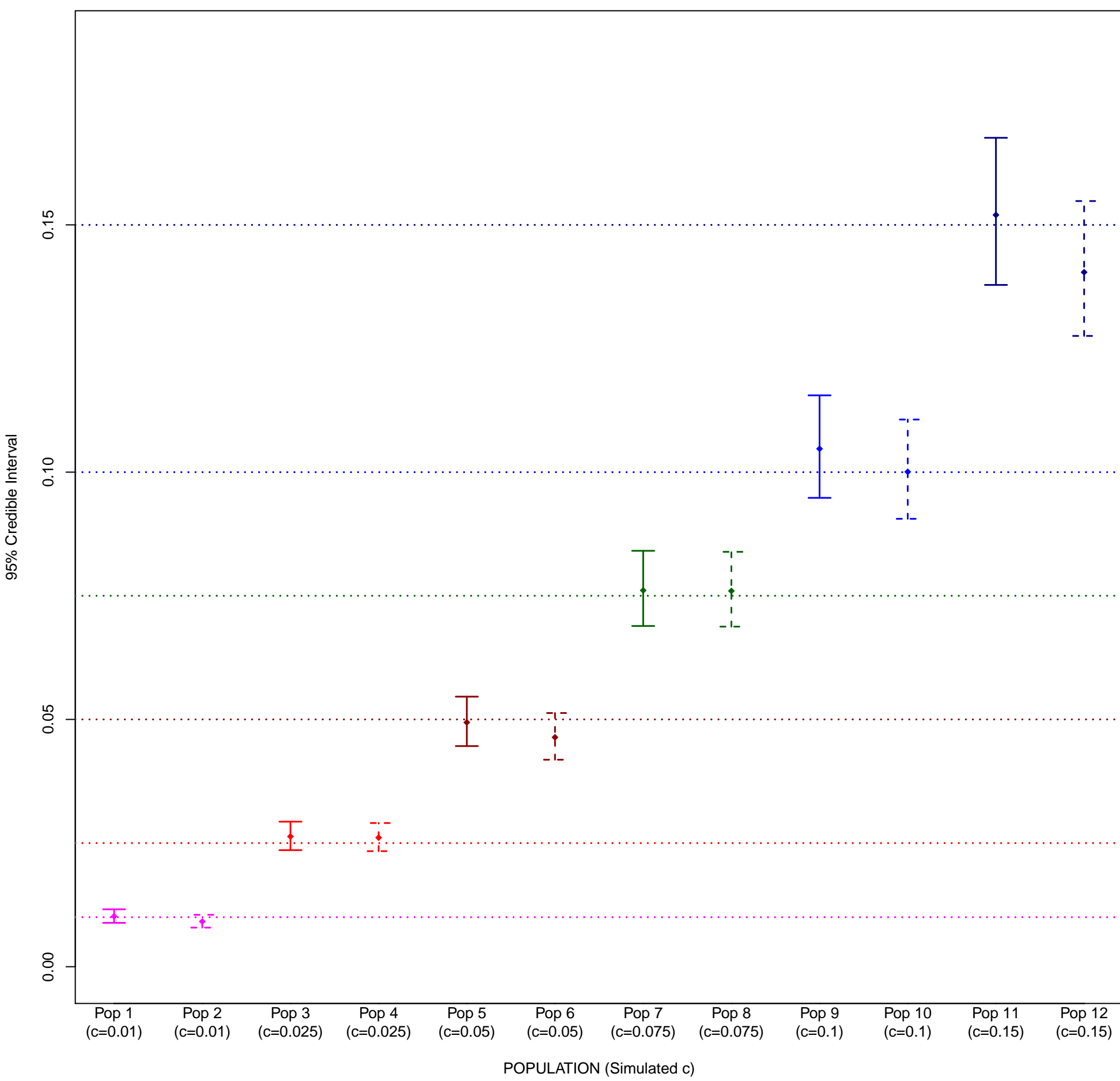

**C) DS2 analyzed with 1**

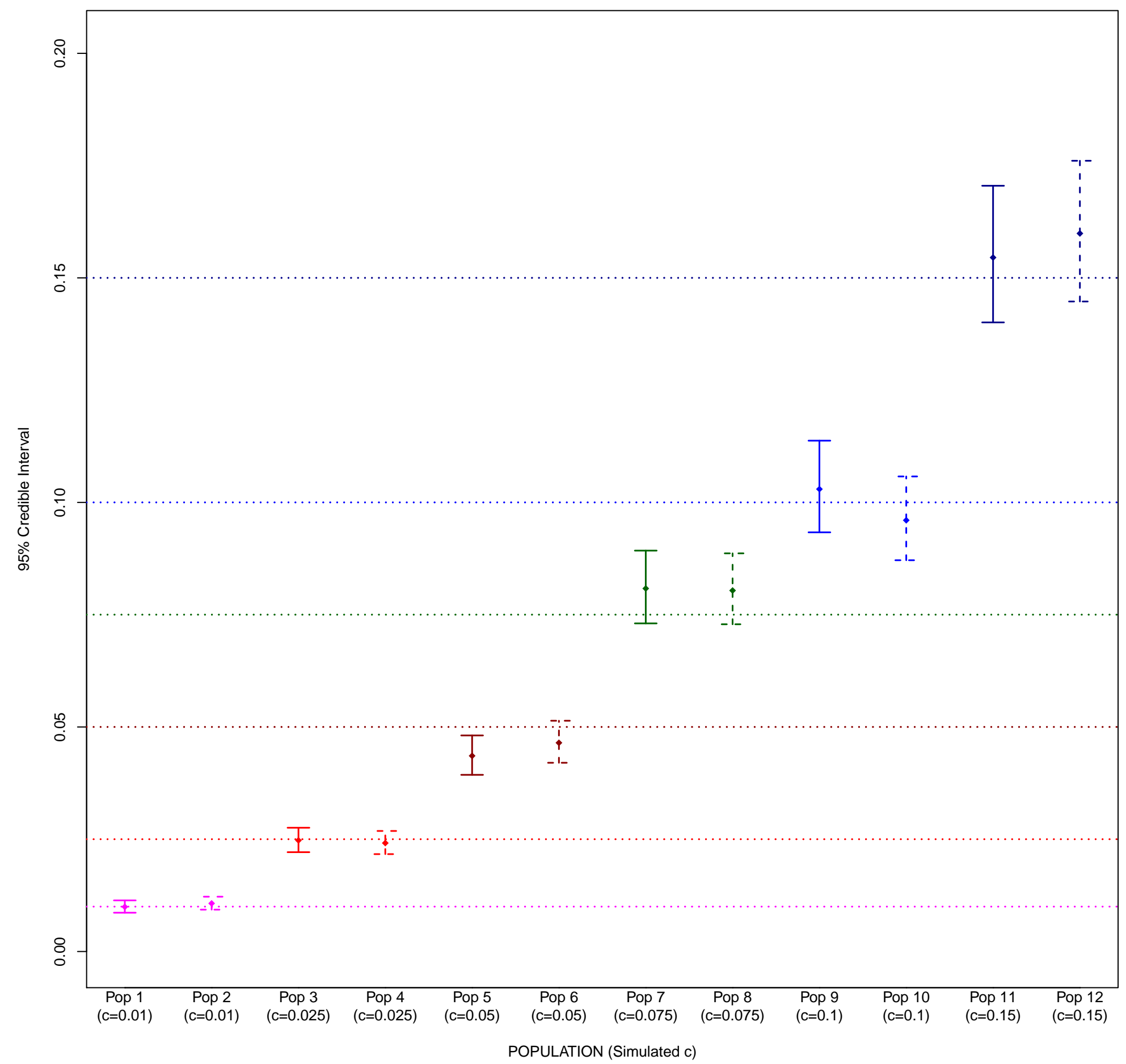

**B) DS1 analyzed with Model 2**

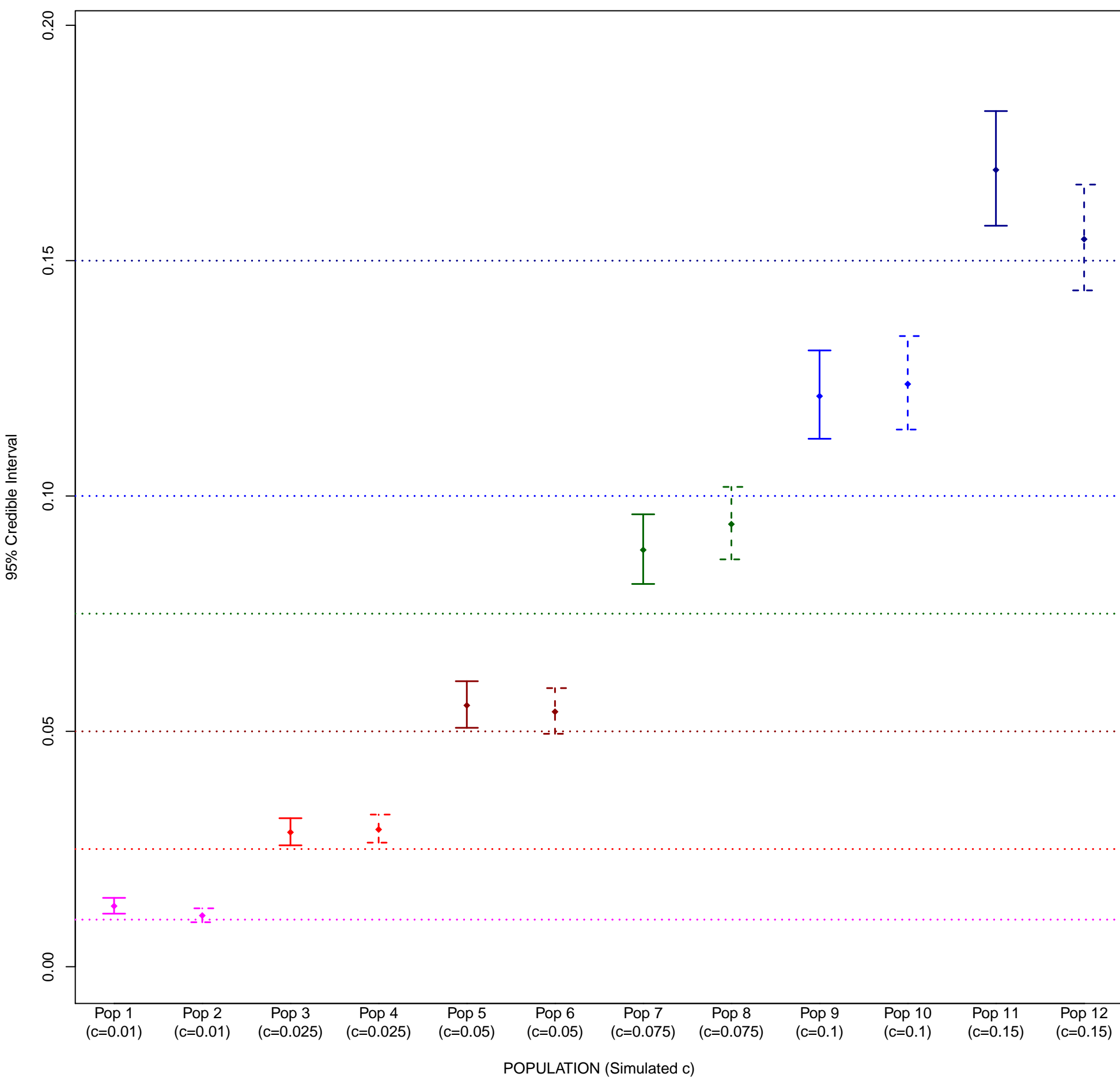

**D) DS2 analyzed with Model 2**

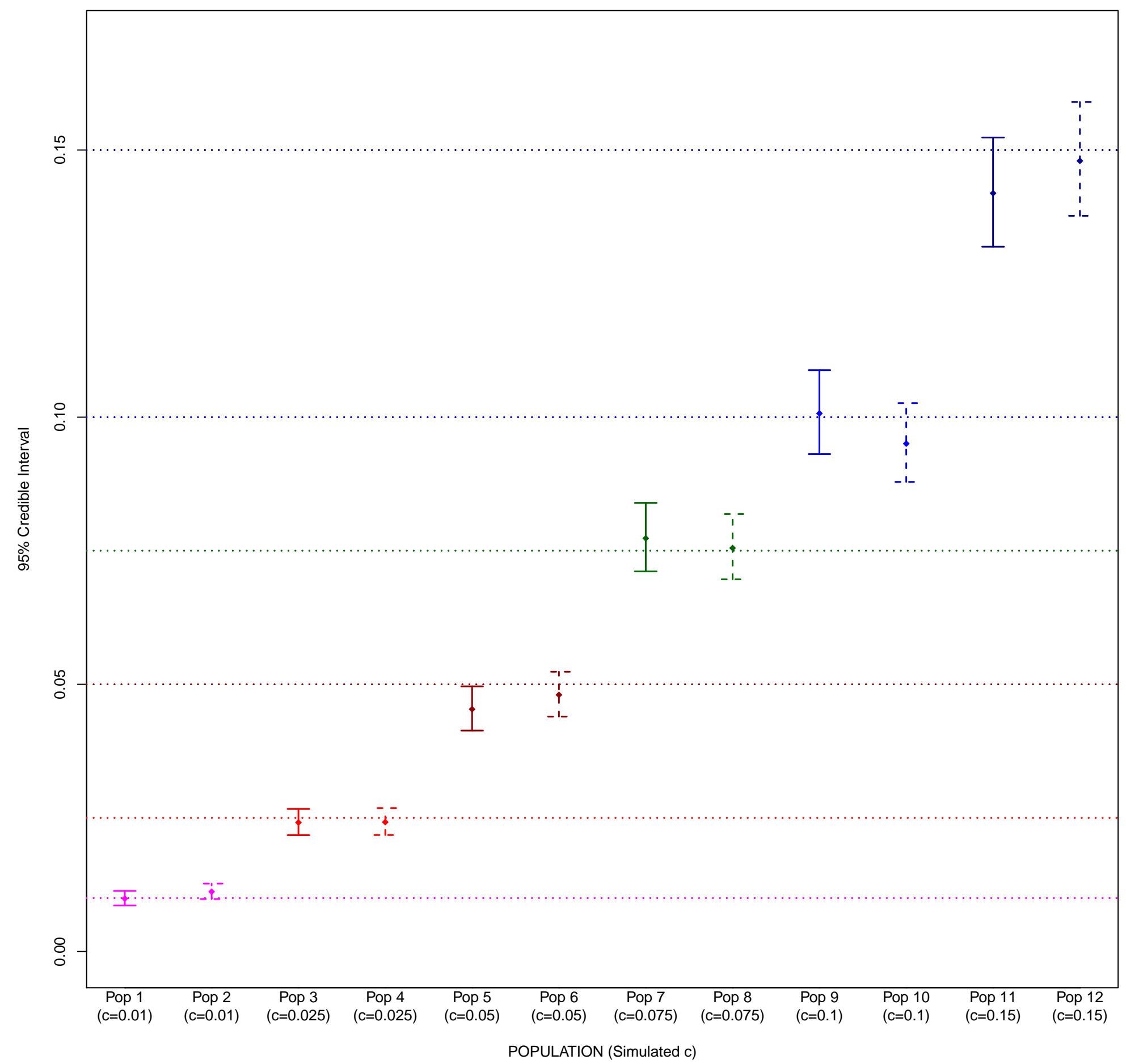

Supplement: Figure S1 — 95% equal tail Credible Interval for the differentiation parameter c obtained after analyzing two data sets DS1 and DS2 simulated respectively under inference model 1 and 2. The two simulated data sets consist of 1,000 SNPs and 12 populations with the following simulated value of c : c1 = c2 = 0.01, c3 = c4 = 0.025, c5 = c6 = 0.05, c7 = c8 = 0.075, c9 = c10 = 0.1 and c11 = c12 = 0.15. A) Data set DS1 analyzed with model 1, B) Data set DS1 analyzed with model 2, C) Data set DS2 analyzed with model 2, D) Data set DS2 analyzed with model 2. (0.03 MB PDF) [file pone.0011913.s006.pdf]

A) Analysis with model 1

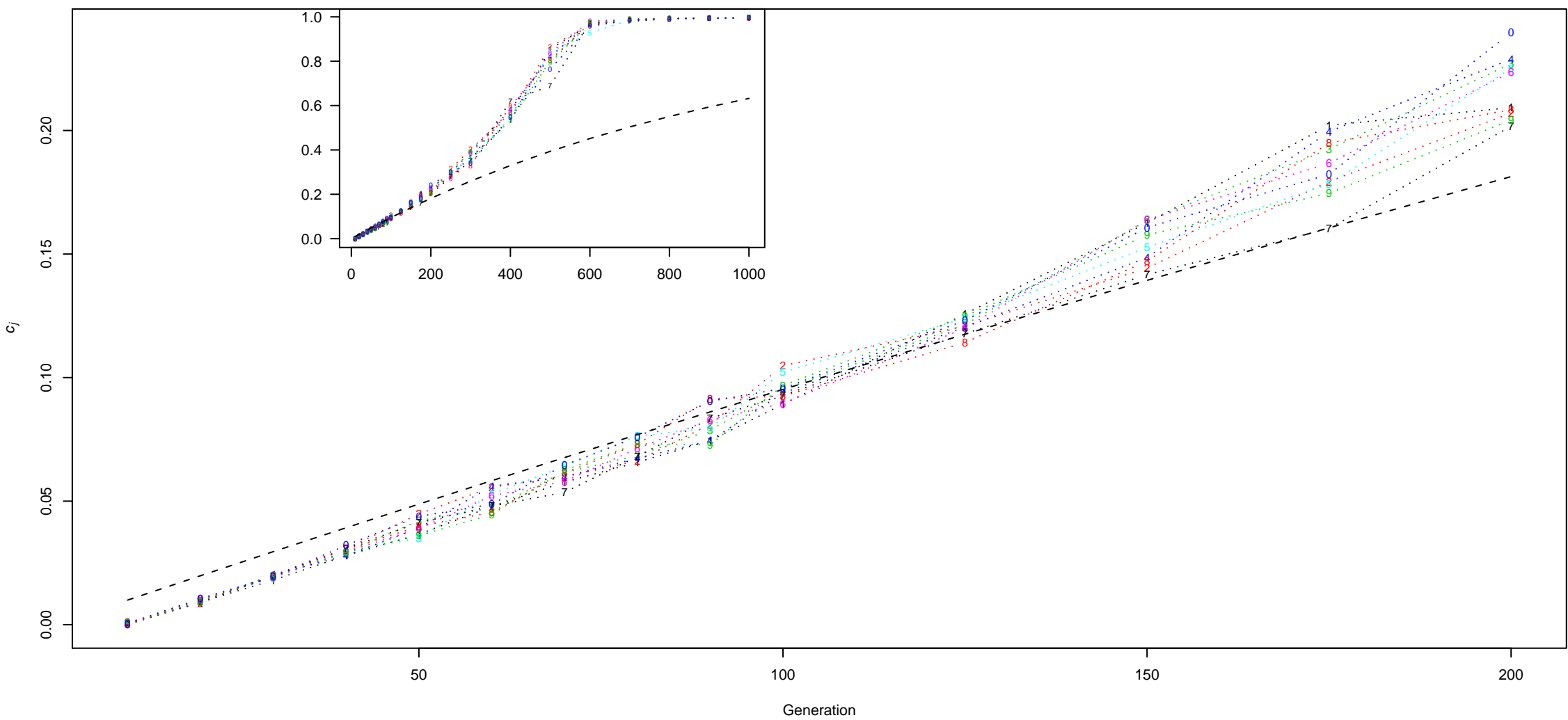

B) Analysis with model 2

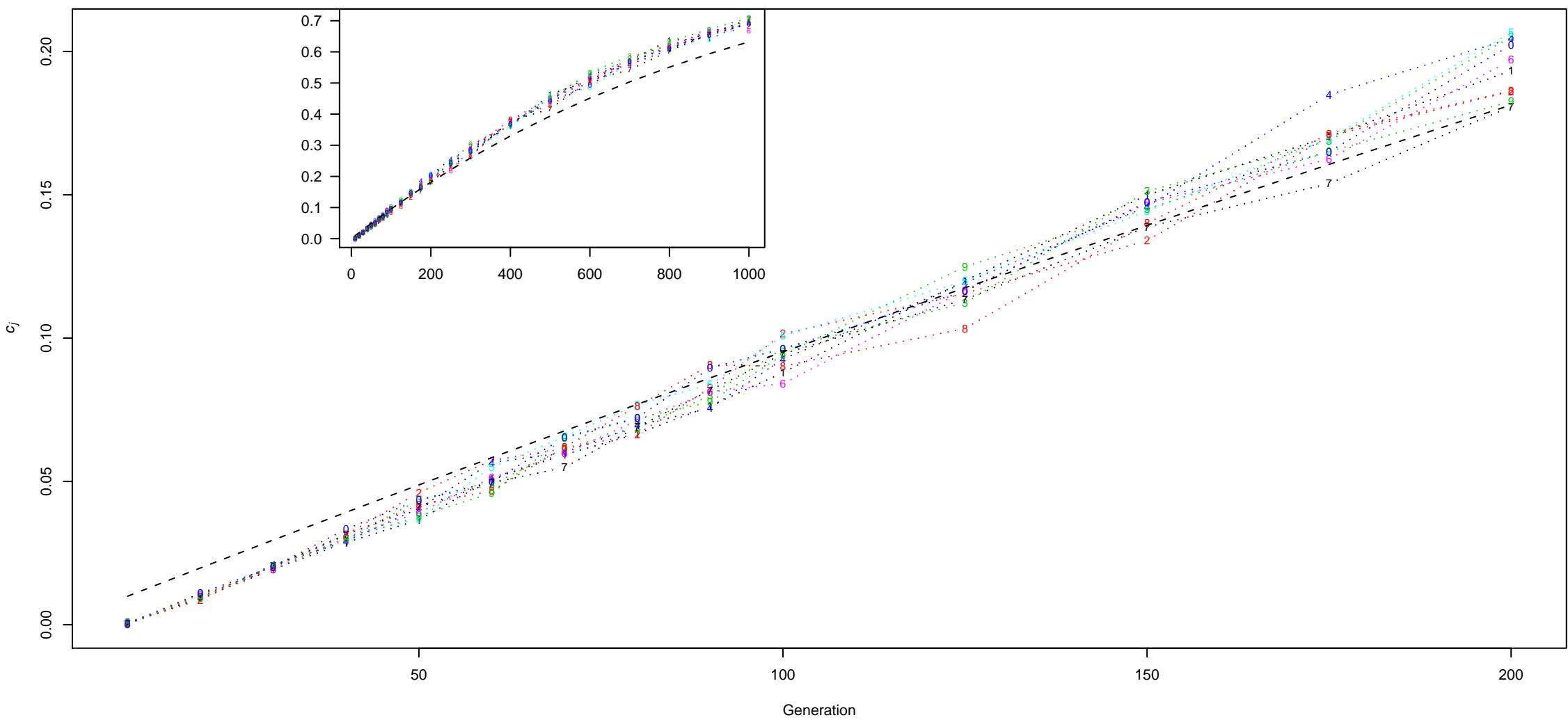

Supplement: Figure S2 — Estimates of c for 17 data sets simulated under a pure-drift demographic model. Allele counts for 1,000 (neutral) SNPs were simulated for 10 populations and for 23 different times (measured in number of discrete generations) after divergence (T = 10, T = 20, T = 30, T = 40, T = 50, T = 60, T = 70, T = 80, T = 90, T = 100, T = 125, T = 150, T = 175, T = 200, T = 250, T = 300, T = 400, T = 500, T = 600, T = 700, T = 800, T = 900 and T = 1000). The resulting data sets were analyzed using both model 1 (A) and model 2 (B). Resulting estimates (mean of the posterior distribution) are plotted against the corresponding simulated time (the different number representing the population label) and are connected by a line. The grey dashed line represents the expected FST value (see Methods). (0.07 MB PDF) [file pone.0011913.s007.pdf]

**Cj=0.02 Pi=0.05**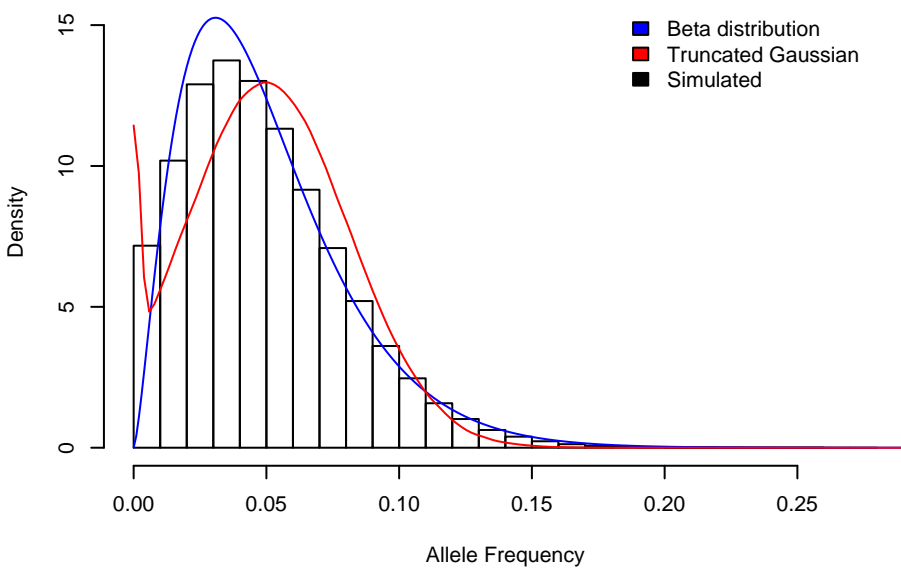**Cj=0.02 Pi=0.25**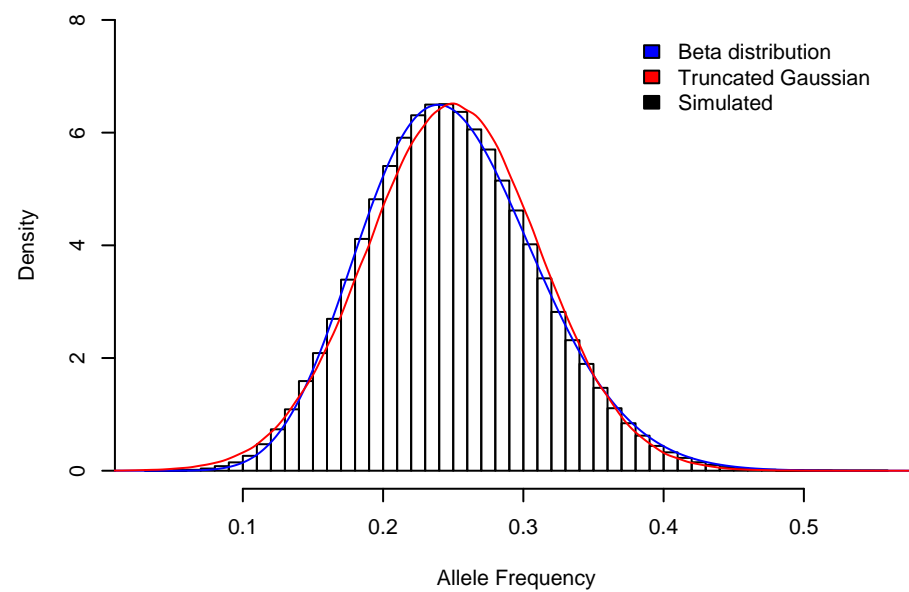**Cj=0.02 Pi=0.5**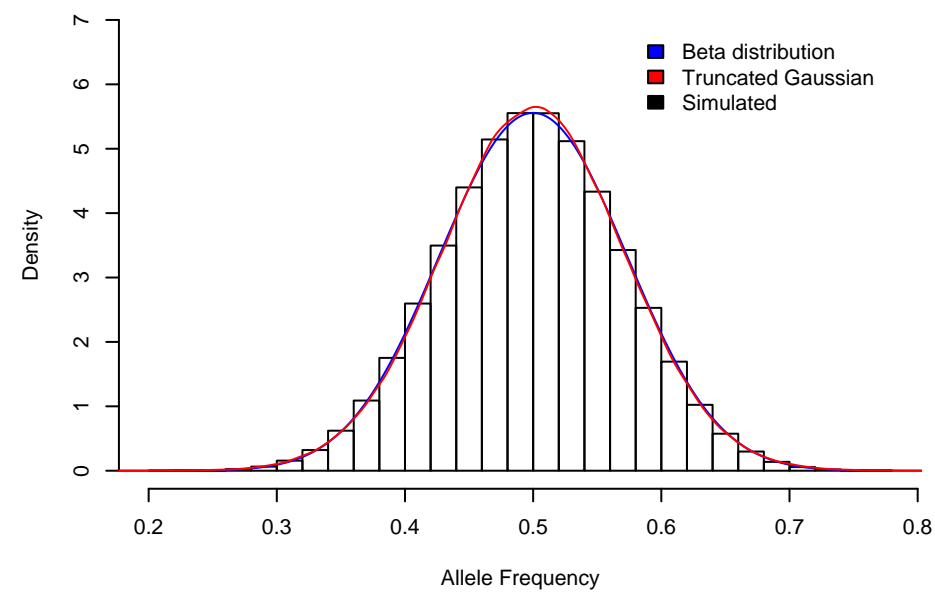**Cj=0.1 Pi=0.05**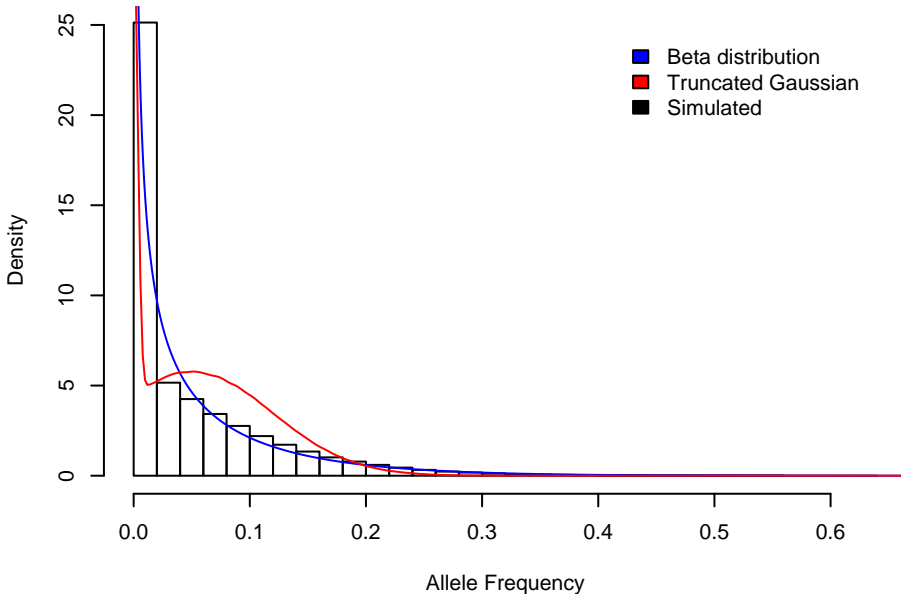**Cj=0.1 Pi=0.25**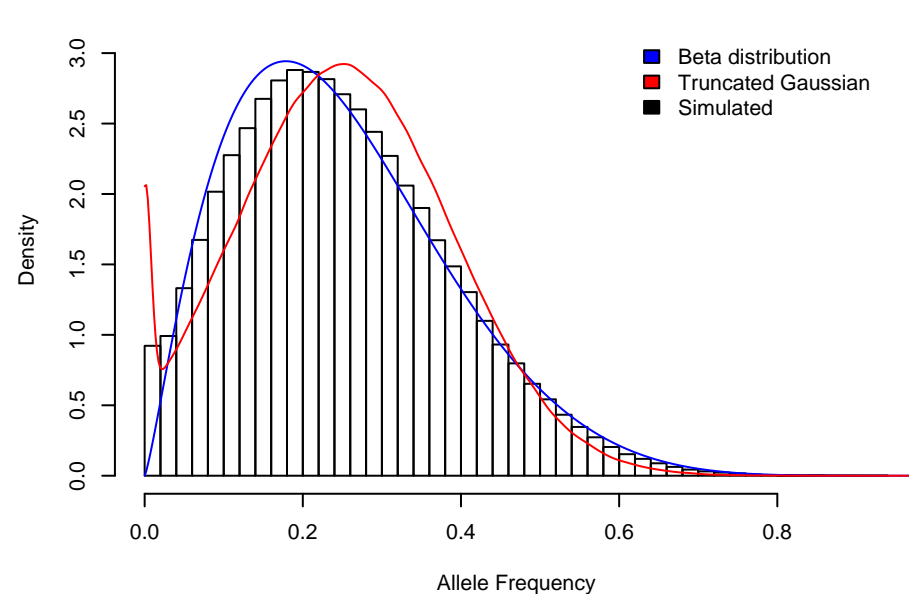**Cj=0.1 Pi=0.5**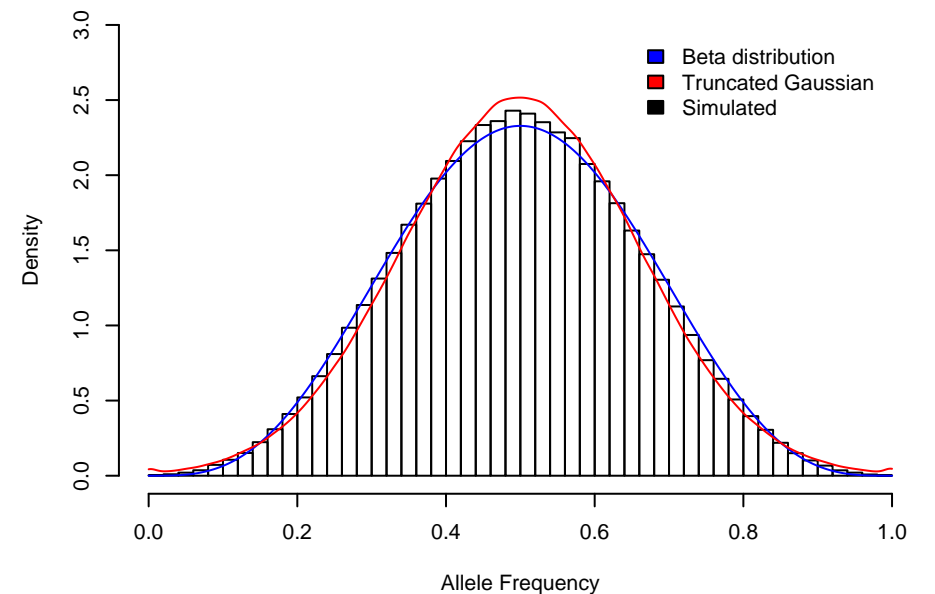**Cj=0.25 Pi=0.05**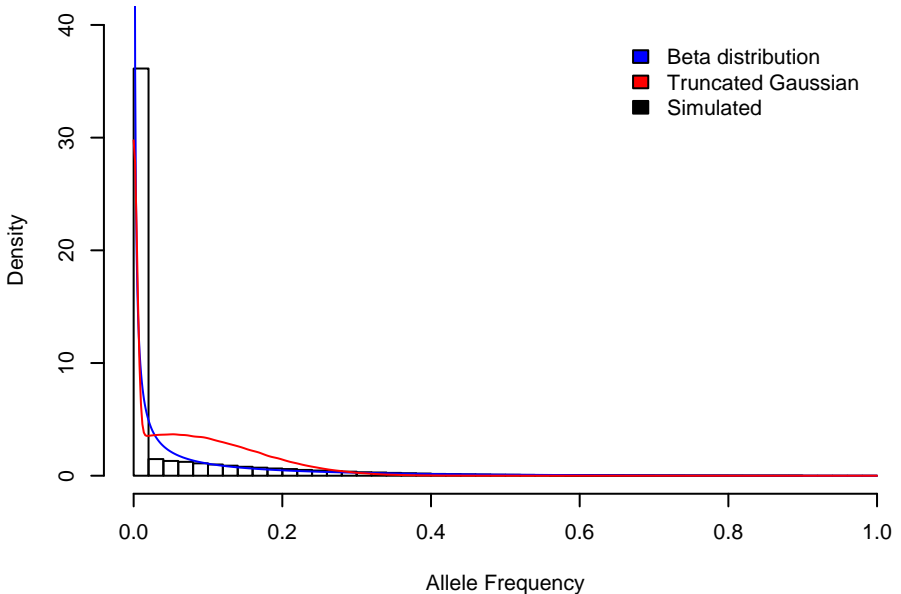**Cj=0.25 Pi=0.25**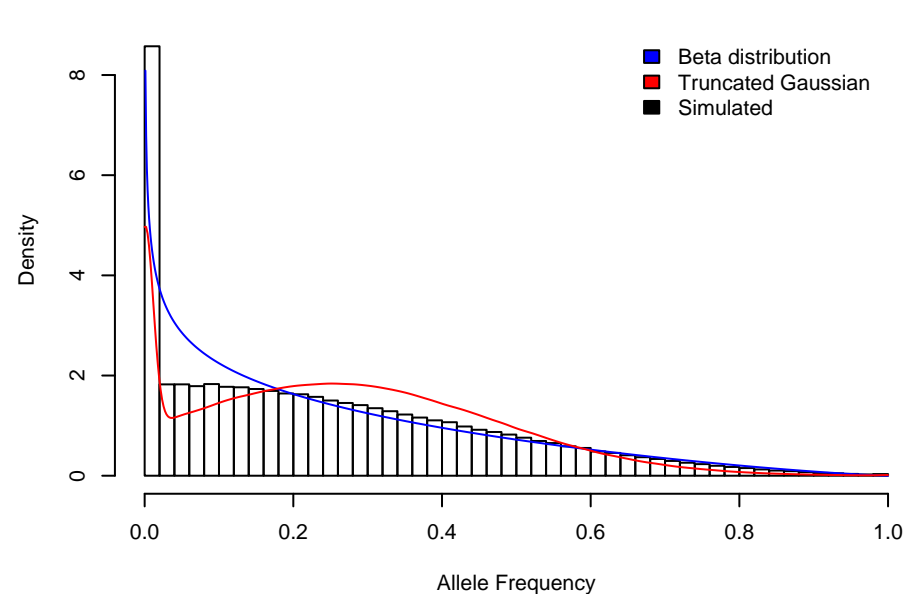**Cj=0.25 Pi=0.5**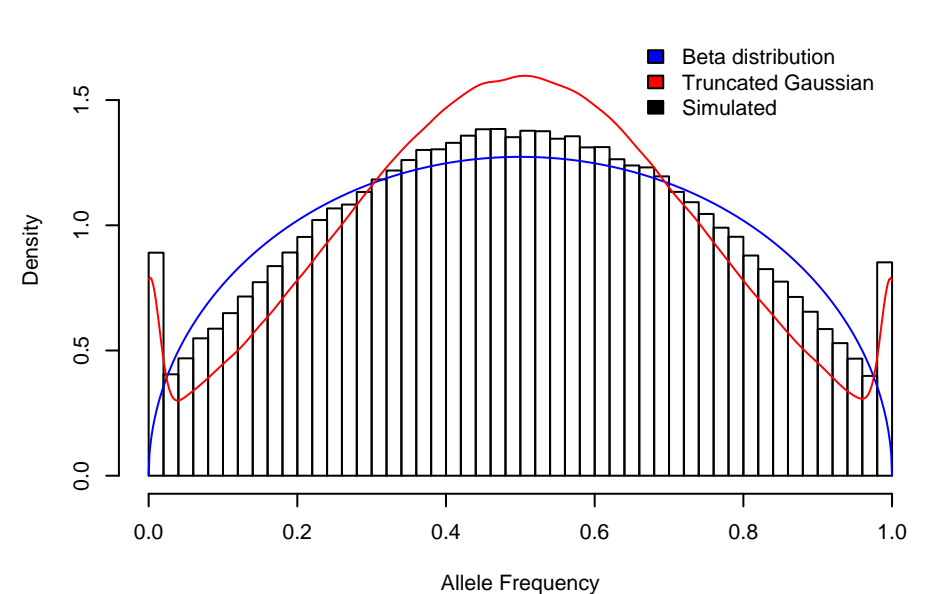**Cj=0.5 Pi=0.05**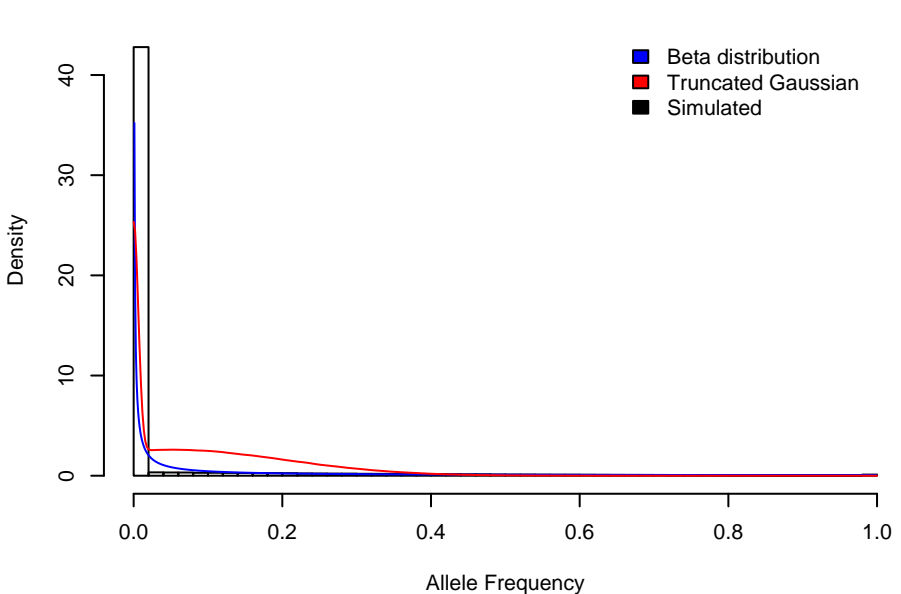**Cj=0.5 Pi=0.25**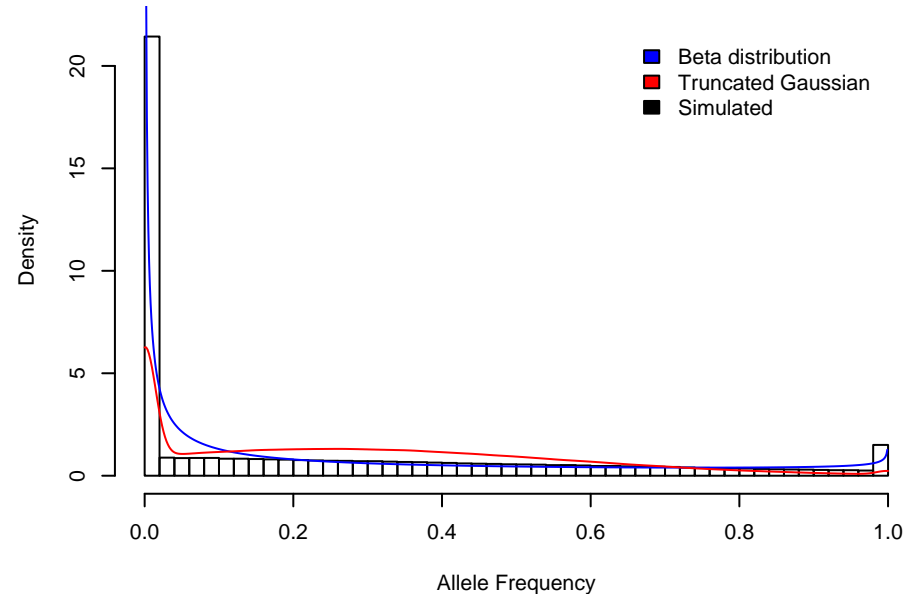**Cj=0.5 Pi=0.5**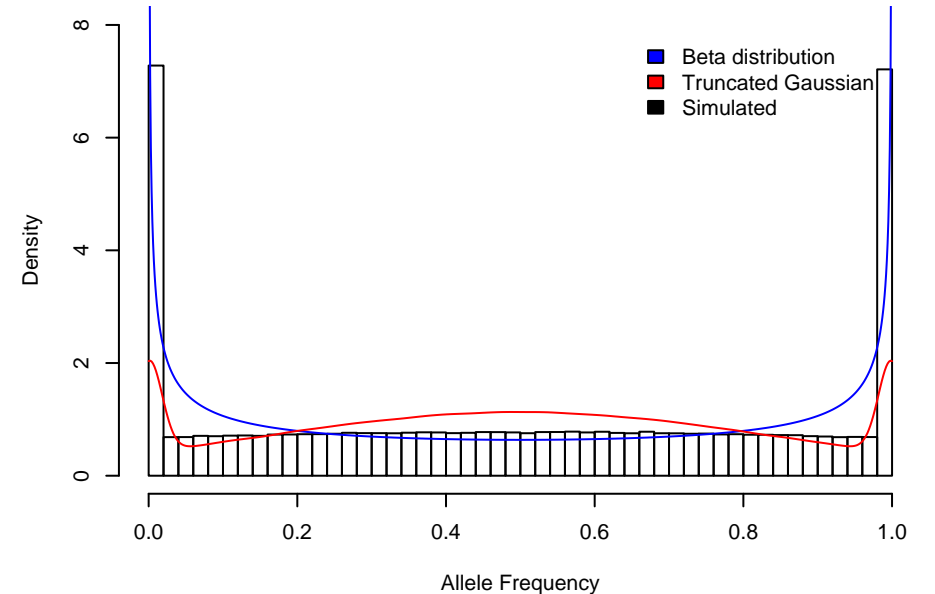

Supplement: Figure S3 — Allele frequency distribution within a population of constant (haploid) effective size (Ne = 500) evolving during T discrete generations (T = log(1−c)/log(1−1/Ne) where c is a measure a population differentiation) as a function of the initial allele frequency (Pi). For each case investigated, a histogram of 1,000,000 simulated values is plotted together with the corresponding densities from model 1 (truncated Gaussian in blue with probability masses in 0 and 1) and model 2 (Beta distribution). Note that an exact derivation of the corresponding distribution has been derived using a forward-time diffusion approach [24]. (0.32 MB PDF) [file pone.0011913.s008.pdf]

**A) Analysis with model 1**

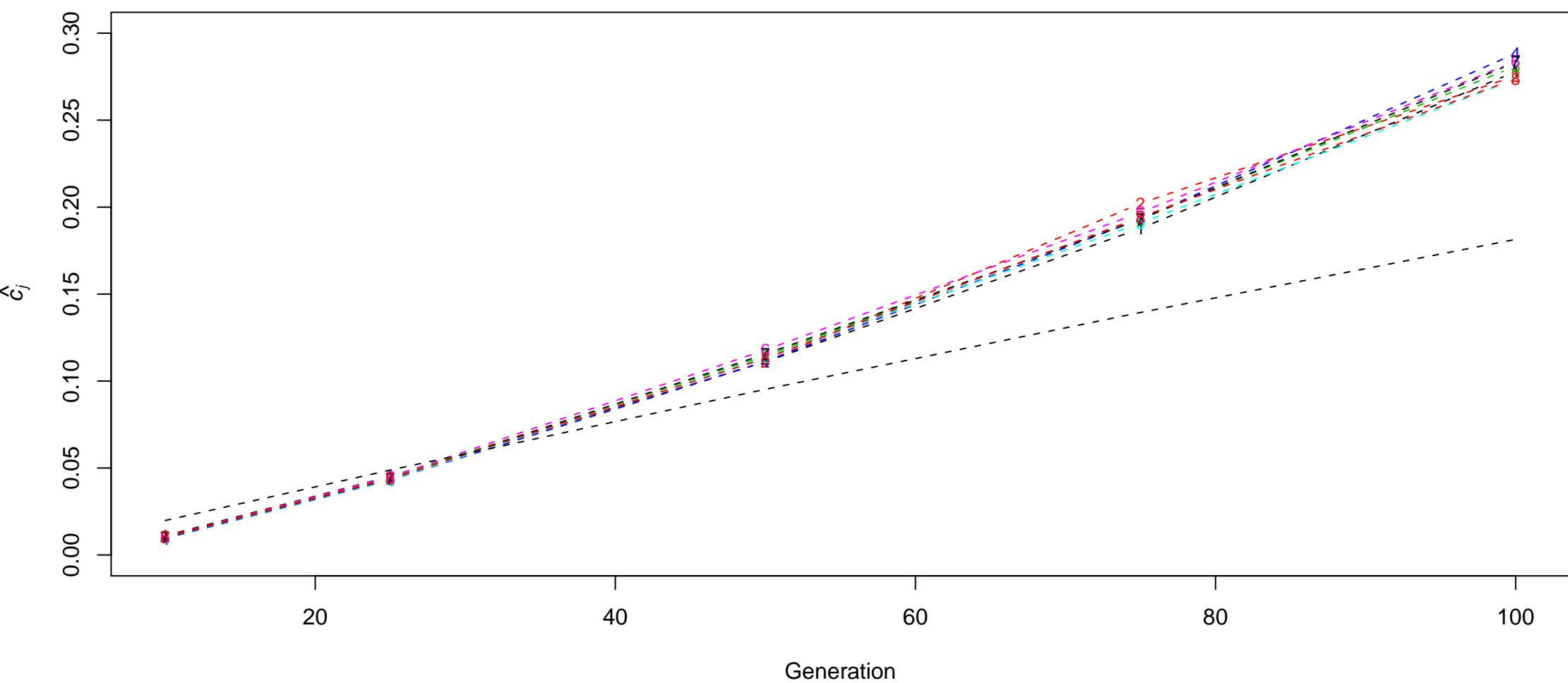

**B) Analysis with model 2**

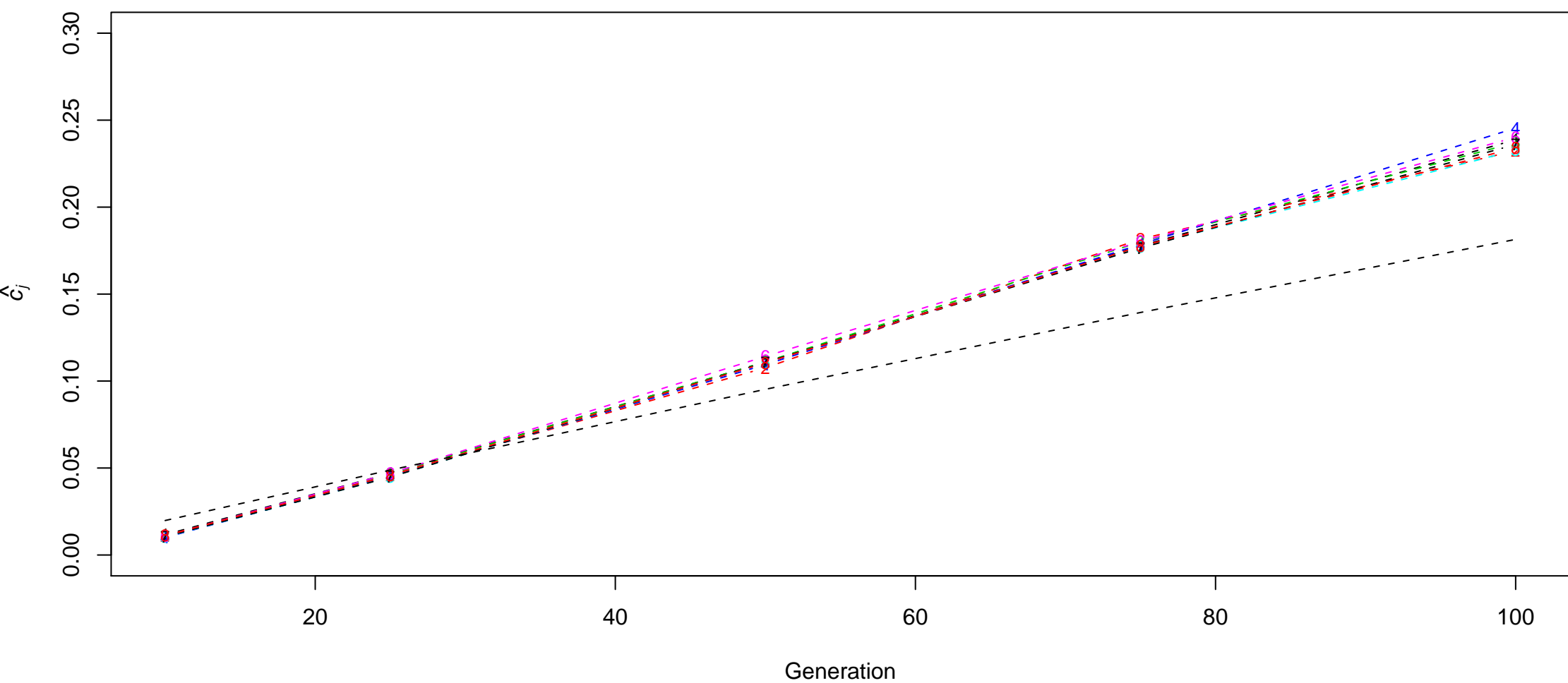

Supplement: Figure S4 — Estimates of c for five data sets simulated under a pure-drift demographic model. Allele counts for 10,000 SNPs (8,500 neutral SNPs, 750 subjected to positive selection and 750 to balancing selection) were simulated for 8 populations and for 5 different times (measured in number of discrete generations) after divergence (T = 10, T = 25, T = 50, T = 75 and T = 100). The five resulting data sets were analyzed using both model 1 (A) and model 2 (B). Resulting estimates (mean of the posterior distribution) are plotted against the corresponding simulated time (the different number representing the population label) and are connected by a line. The grey dashed line represents the expected FST value (see Methods). (0.01 MB PDF) [file pone.0011913.s009.pdf]

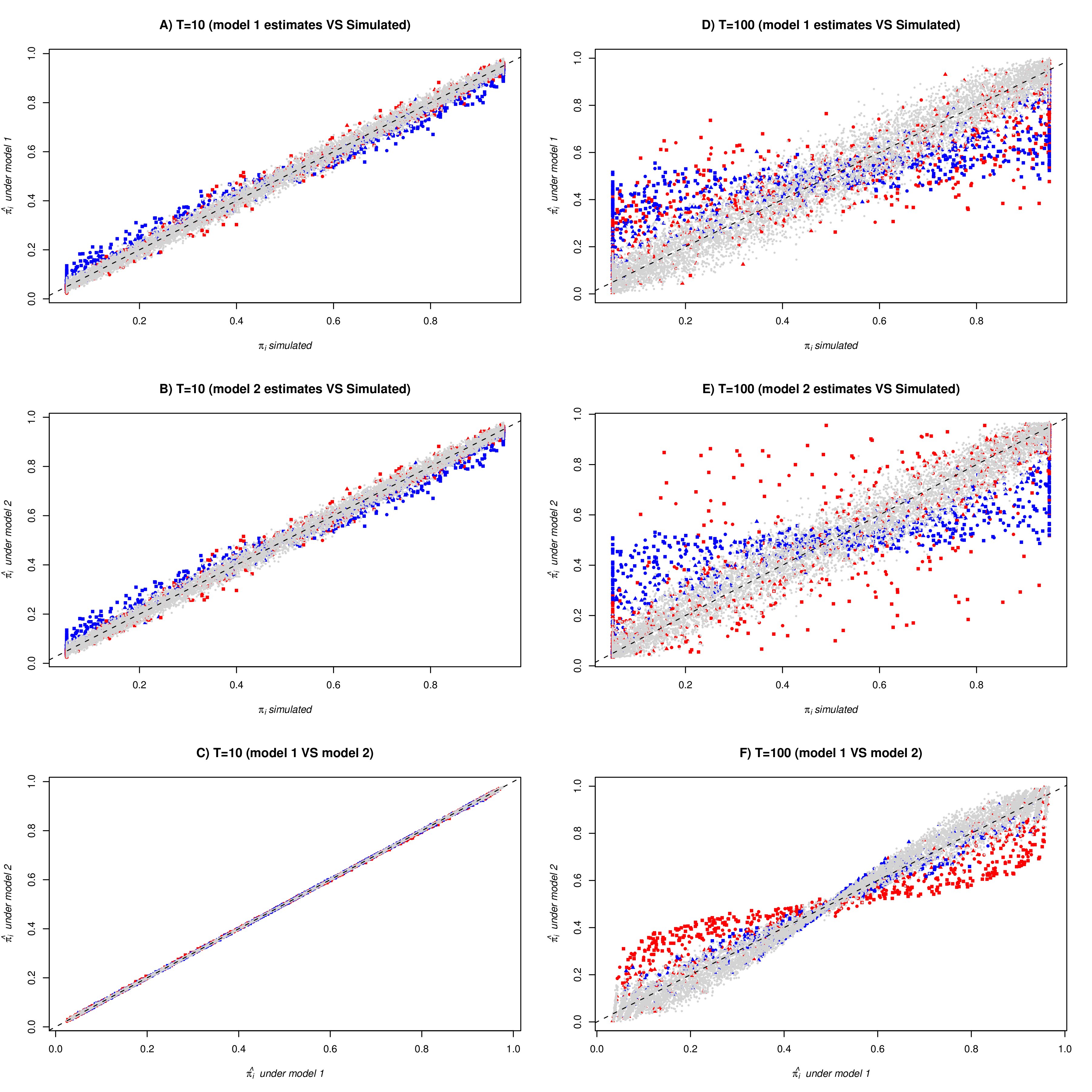

Supplement: Figure S5 — Robustness of the estimates (mean of the posterior distribution) of the ancestral (reference) allele frequency πi. Two data sets (T = 10 and T = 100) consisting in genotyping data for 10,000 SNPs (8,500 neutral SNPs, 750 subjected to positive selection and 750 subjected to balancing selection) on 8 populations were analyzed with model 1 and model 2 (see text). For each data set, three plots are shown: i) estimates obtained under model 1 against (true) simulated values (A with T = 10 and D with T = 100), ii) estimates obtained under model 2 against (true) simulated values (B with T = 10 and E with T = 100) and iii) estimates obtained under model 1 against estimates obtained under model 2 (C with T = 10 and E with T = 100). Neutral SNPs are plotted in grey while SNPs subjected to positive (respectively balancing) selection are plotted in red (respectively blue). In addition, the simulated coefficients of selection are represented by a triangle (s = 0.02), a circle (s = 0.05) or a square (s = 0.10). (1.06 MB JPG) [file pone.0011913.s010.jpg]

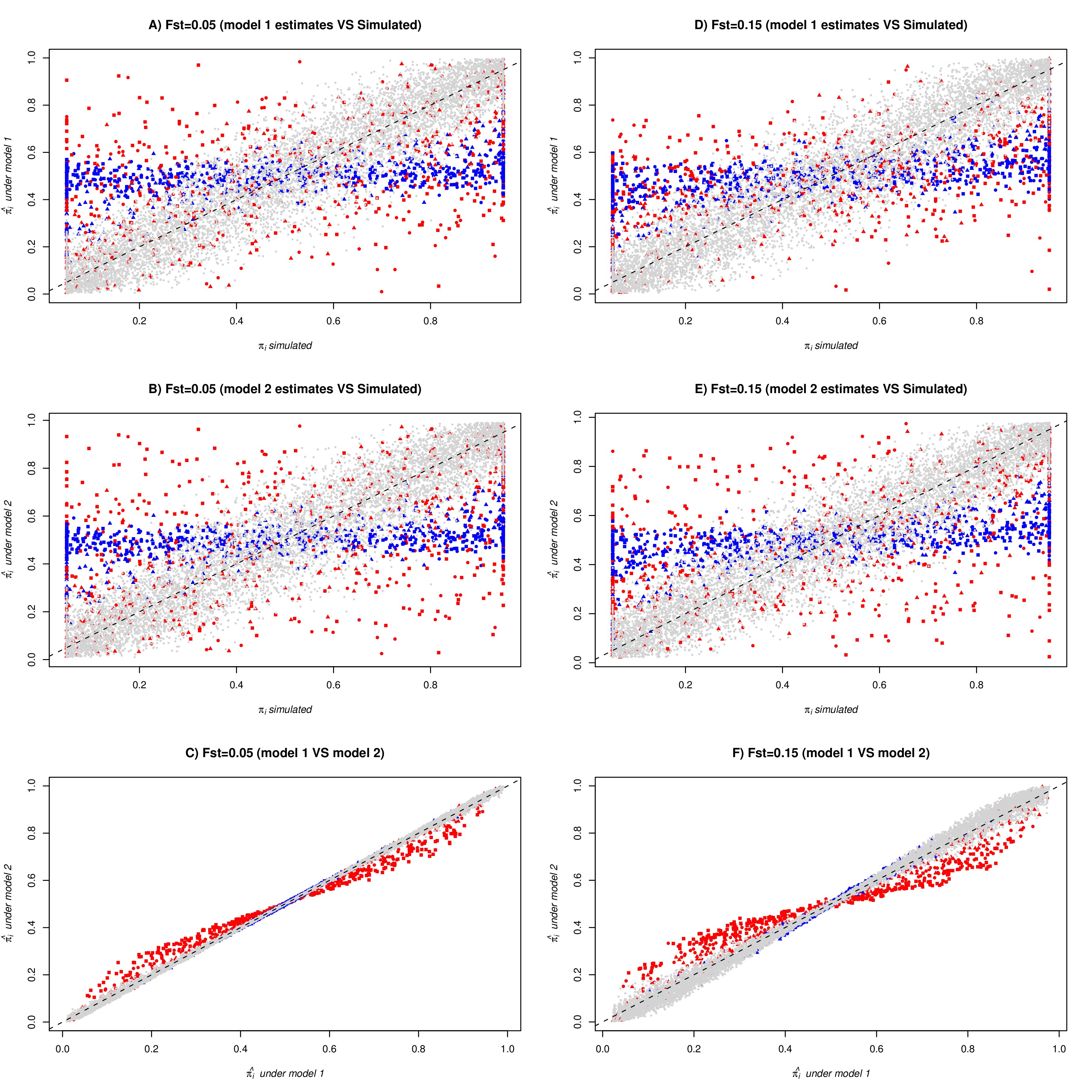

Supplement: Figure S6 — Robustness of the estimates (mean of the posterior distribution) of the (reference) allele frequency πi in the gene pool. Two data sets (FST = 0.05 and FST = 0.15) consisting in genotyping data for 10,000 SNPs (8,500 neutral SNPs, 750 subjected to positive selection and 750 subjected to balancing selection) on 8 populations were analyzed with model 1 and model 2 (see text). For each data set, three plots are represented: i) estimates obtained under model 1 against (true) simulated values (A with FST = 0.05 and D with FST = 0.15), ii) estimates obtained under model 2 against (true) simulated values (B with FST = 0.05 and E with FST = 0.15) and iii) estimates obtained under model 1 against estimates obtained under model 2 (C with FST = 0.05 and E with FST = 0.15). Neutral SNPs are plotted in grey while SNPs subjected to positive (respectively balancing) selection are plotted in red (respectively blue). In addition, the simulated coefficients of selection are represented by a triangle (s = 0.02), a circle (s = 0.05) or a square (s = 0.10). (1.51 MB JPG) [file pone.0011913.s011.jpg]
